# Supervised learning with word embeddings derived from PubMed captures latent knowledge about protein kinases and cancer

**DOI:** 10.1093/nargab/lqab113

**Published:** 2021-12-08

**Authors:** Vida Ravanmehr, Hannah Blau, Luca Cappelletti, Tommaso Fontana, Leigh Carmody, Ben Coleman, Joshy George, Justin Reese, Marcin Joachimiak, Giovanni Bocci, Peter Hansen, Carol Bult, Jens Rueter, Elena Casiraghi, Giorgio Valentini, Christopher Mungall, Tudor I Oprea, Peter N Robinson

**Affiliations:** The Jackson Laboratory for Genomic Medicine, Farmington, CT 06032, USA; The Jackson Laboratory for Genomic Medicine, Farmington, CT 06032, USA; AnacletoLab, Dipartimento di Informatica, Università degli Studi di Milano, Italy; AnacletoLab, Dipartimento di Informatica, Università degli Studi di Milano, Italy; The Jackson Laboratory for Genomic Medicine, Farmington, CT 06032, USA; The Jackson Laboratory for Genomic Medicine, Farmington, CT 06032, USA; University of Connecticut Health Center, Department of Genetics and Genome Sciences, Farmington, CT 06030, USA; The Jackson Laboratory for Genomic Medicine, Farmington, CT 06032, USA; Division of Environmental Genomics and Systems Biology, Lawrence Berkeley National Laboratory, Berkeley, CA 94710, USA; Division of Environmental Genomics and Systems Biology, Lawrence Berkeley National Laboratory, Berkeley, CA 94710, USA; Department of Internal Medicine and UNM Comprehensive Cancer Center, UNM School of, Medicine, Albuquerque, NM 87102, USA; The Jackson Laboratory for Genomic Medicine, Farmington, CT 06032, USA; The Jackson Laboratory for Mammalian Genetics, Bar Harbor, ME 04609, USA; The Jackson Laboratory for Mammalian Genetics, Bar Harbor, ME 04609, USA; AnacletoLab, Dipartimento di Informatica, Università degli Studi di Milano, Italy; AnacletoLab, Dipartimento di Informatica, Università degli Studi di Milano, Italy; Division of Environmental Genomics and Systems Biology, Lawrence Berkeley National Laboratory, Berkeley, CA 94710, USA; Department of Internal Medicine and UNM Comprehensive Cancer Center, UNM School of, Medicine, Albuquerque, NM 87102, USA; The Jackson Laboratory for Genomic Medicine, Farmington, CT 06032, USA; Institute for Systems Genomics, University of Connecticut, Farmington, CT 06032, USA

## Abstract

Inhibiting protein kinases (PKs) that cause cancers has been an important topic in cancer therapy for years. So far, almost 8% of >530 PKs have been targeted by FDA-approved medications, and around 150 protein kinase inhibitors (PKIs) have been tested in clinical trials. We present an approach based on natural language processing and machine learning to investigate the relations between PKs and cancers, predicting PKs whose inhibition would be efficacious to treat a certain cancer. Our approach represents PKs and cancers as semantically meaningful 100-dimensional vectors based on word and concept neighborhoods in PubMed abstracts. We use information about phase I-IV trials in ClinicalTrials.gov to construct a training set for random forest classification. Our results with historical data show that associations between PKs and specific cancers can be predicted years in advance with good accuracy. Our tool can be used to predict the relevance of inhibiting PKs for specific cancers and to support the design of well-focused clinical trials to discover novel PKIs for cancer therapy.

## INTRODUCTION

Protein phosphorylation is one of the most important post-translational modifications. The human genome encodes 538 protein kinases (PKs), many of which are associated with cancer initiation or progression. PKs transfer a γ-phosphate group from ATP to serine, threonine, or tyrosine residues; the genome encodes roughly 200 phosphatases that remove a phosphate group from a protein. Protein phosphorylation and dephosphorylation are involved in virtually every basic cellular process including proliferation, cell cycle, apoptosis, motility, growth and differentiation. Many PKs promote cell proliferation, survival, and migration, and misregulation of kinase activity is a common feature of oncogenesis (1–3). Molecularly targeted cancer therapies are rapidly growing in importance for the treatment of many types of cancer. Many targeted therapies, including small-molecule kinase inhibitors and monoclonal antibodies, act as PK inhibitors (PKIs). Since the introduction of the initial PKI in the 1980s, at least 37 PKIs have received FDA approval for cancer therapy and over 150 kinase-targeted drugs are in clinical trials (3).

PKIs are not equally effective for all cancer types; instead, specific characteristics of each tumor, including genetics, tumor microenvironment, drug resistance, and pharmacogenomics determine how useful a compound will be in the treatment of a given cancer. Factors including whether a particular kinase exhibits activating mutations in a given cancer, or whether downstream targets of the kinase are mutated strongly influence the susceptibility of a cancer to a given PKI. Characteristics of pathways related to those mutated in a given cancer can also influence response to targeted treatment (4). In addition, most PKIs target more than one protein with a range from highly to poorly selective (5). It is, therefore, not always possible to reliably predict whether a given PKI will be efficacious against a given type of cancer. For instance, imatinib, which targets BCR-ABL, c-Abl, PDGFR and c-Kit, was found not be effective in uveal melanoma despite high expression of KIT, an unexpected finding that was interpreted to be related to the lack of ERK phosphorylation in these tumors (6).

In this work, we pose the question of whether one can use knowledge latent in the published literature to predict whether inhibition of a given PK is an effective treatment of a cancer. Correct predictions could be used to prioritize clinical trials of a cancer with PKIs that target the PK in question. In particular, our aim is to exploit the large corpus of clinical text data available in PubMed abstracts to discover novel associations between PKs and cancer, leveraging natural language processing approaches based on word embedding that have been successfully applied to text analysis, representation, and classification tasks (7). On historical data, we achieved an area under the receiver operating characteristic (ROC) curve (AUROC) of up to 86.3% for predicting successful trials of all phases and up to 96.3% for predicting successful phase IV trials. Predictions based on PubMed data through 2020 revealed 2979 of 325 494 untested PK-cancer pairs (0.92%) had above-threshold probabilities.

## MATERIALS AND METHODS

### Text normalization and preprocessing

We developed a software package called marea (**m**area **a**damantly **r**esists **e**gregious **a**cronyms) that implements all necessary natural language processing (NLP) steps to prepare the titles and abstracts of PubMed articles as input for word embedding algorithms. marea filters PubMed articles for relevance and applies PubTator Central (8) concept recognition to the titles and abstracts of relevant articles. After concept replacement, the final phase eliminates punctuation and stop words and reduces the vocabulary size.

#### Filtering relevant PubMed articles

NCBI’s FTP site makes available gzipped XML files containing titles, abstracts, and metadata for all PubMed articles. marea downloads the annual baseline and daily update files, and parses them to extract the fields of interest for each article: PubMed ID, MeSH descriptors (if any), keywords (if any), and year of publication. For entries that have multiple dates with different years, the earliest one is recorded. To select articles for a particular search, the marea user provides a set of high-level MeSH descriptor ids. The MeSH descriptors defining the scope of the research described herein were D009369 (Neoplasms) and D011494 (Protein Kinases). Any article marked with at least one of these descriptors or any subcategory of these descriptors is considered relevant. An article is also judged relevant if it has a keyword that matches a label or synonym of the search descriptors or their subcategories. Some PubMed articles have neither MeSH descriptors nor keywords; these cannot match the search. Any article that lacks an abstract is deemed irrelevant regardless of its MeSH descriptors or keywords.

#### Concept replacement

The original word2vec method (9,10) operates on individual words (tokens). However, many medical concepts span multiple tokens. For instance, *non-small-cell lung carcinoma* would be treated by word2vec as three or five tokens (depending on how the hyphen is handled in preprocessing), but it represents a single medical concept. For this reason, recent approaches collapse multiword concepts into a single token prior to embedding by replacing the multiword concepts with a single concept id (11). For instance, *non-small-cell lung carcinoma* can be replaced by its MeSH id D002289.

PubTator Central from the National Center for Biotechnology Information (National Library of Medicine) offers data for concept recognition in PubMed articles. Annotated categories include chemicals, diseases, genes, cell lines, SNPs and species, as well as other categories marea does not track, such as DNAMutation and ProteinMutation. Using PubTator Central character offsets, our software replaces each phrase recognized in the title or abstract with the identifier of the corresponding concept. Diseases and chemical names are normalized to MeSH ids, genes and proteins to NCBI Gene ids, cell lines to Cellosaurus (12), SNPs to dbSNP RS ids and species to NCBI Taxonomy ids. The one exception is the human species, NCBI taxon 9606, which we decided to skip. PubTator Central annotations would have substituted 9606 for *man*, *woman*, *boy*, *girl*, *father*, *mother*, *patient* and similar words. We chose to preserve the distinctions of gender and age expressed in terms for humans, as these factors are significant in the medical context.

#### Text preprocessing

After concept replacement, marea cleans up the text of PubMed titles and abstracts to make it more suitable for word embedding. The tokenizer deletes all punctuation symbols, including hyphens and underscores within words: the parts of a compound word become separate tokens. marea removes stop words, whether lowercase or capitalized. Uppercase acronyms of length ≥2, even those that coincide with stop words, are not changed. For example, the acronym *ALL* (acute lymphocytic leukemia) is retained while *all* and *All* are eliminated. We started with the stop word list for English in the Natural Language Toolkit (nltk version 3.5) Python library (13) and added some new stop words. Any letter of the alphabet that occurs as a single-character token is a stop word. To further reduce the size of the vocabulary, tokens that remain after stop word removal are lemmatized with the WordNet (14) lemmatizer from nltk. The lemmatizer reduces words to their base form, for example plural nouns are simplified to the singular (unlike stemming, lemmatizing a word always returns a complete word, not a truncated word stem). The last step of text preprocessing converts everything to lowercase, to avoid near-duplicate embeddings for upper-, lower- and mixed-case forms of the same word.

### Word embedding

The word embedding method based on the word2vec algorithm is performed on the preprocessed corpus to embed words to vectors. We used the EMBeddInG GENerator (embiggen), a Python 3 software library developed by our group for word embedding based on word2vec and node embedding based on the node2vec algorithm (15). In the current project, the skip-gram model was used for word2vec with the parameters window size = 5, minimum count (minimum word frequency) = 5, batch size = 128, negative samples = 20 and dimension = 100. Word embedding on the total corpus resulted in embeddings of 293,274 words, each with dimension 100.

### PKIs and their PK targets

The online drug compendium DrugCentral (16) records experimental activities for approved drugs across all major protein target families (including kinases). We extracted the kinase activities from DrugCentral for PKIs. The result of this operation is a list of PKI-PK pairs (PKI2PK), each of which is mapped to an experimental value of affinity (e.g. Ki, IC_50_, etc) in micromolar units and appropriately referenced (when possible) with a PubMed ID (PMID). Moreover, we kept only the PKI2PK pairs having an activity value below 0.03 μM, which is the threshold under which drugs are more likely to act on kinases (17). The last filter that we applied to extract PKI2PK pairs was the number of PKs that are inhibited by a PKI to treat a cancer. For our analysis, we chose PKIs that have an affinity value <0.03 μM and inhibit at most 5 PKs. If a PKI inhibited >5 PKs at this threshold, we chose the top five PKs (n_pk argument in the Python code). Filtering the DrugCentral data by applying the affinity threshold 0.03 μM and a limit of 5 targeted PKs resulted in a list of 226 pairs of PKs and PKIs (Supplementary Material File S1).

For testing (both in the historical experiments and in the de novo predictions), we excluded all PK/cancer pairs derived from any PK–PKI association in the DrugCentral data, regardless of affinity or n_pk.

### Cancers and subtypes

We derived a list of cancers from the Medical Subject Headings (MeSH) thesaurus, yielding a list of 698 neoplasms and their MeSH ids.

### Phase I-Phase IV clinical trials of PKIs for cancer therapy

Clinical trials are typically performed in four standardized phases. A phase I trial is designed to test the safety and pharmacology of a drug. Phase II trials are therapeutic exploratory trials that are conducted in a small number of volunteers with the disease of interest, to answer questions required to prepare a phase III trial including optimal doses, dose frequencies, administration routes, and endpoints. Phase III trials strive to demonstrate or confirm efficacy, often by comparing the intervention of interest with either a standard therapy or a placebo. Additionally, the incidence of common adverse reactions is characterized. Phase IV trials are performed subsequent to initial FDA approval with the goal of identifying less common adverse reactions and in some cases of evaluating a drug in populations different from the original study population (18).

We downloaded the Clinical Trials data from the ClinicalTrials.gov server. Using the Clinical Trials data and the above list of neoplasms, we created a list of neoplasms and PKIs that were used to treat the cancers along with the clinical trial phase, start date, completion date of the clinical trials study, MeSH id for each neoplasm and NCT id for each clinical trial study (Supplementary Material File S2).

### Historical validation: training sets

In order to estimate the performance of our approach, we trained our model on historical snapshots of PubMed and tested the predictive accuracy with Clinical Trials data from subsequent years. For each experiment, we fixed the target year to a specific year and used PubMed abstracts published up to and including this year for word embedding. We constructed the positive and negative training sets described below but limited the Clinical Trials data to entries that were initially registered not later than the target year.

To create the positive training set, we chose all pairs of PKs and cancers where the PKIs were approved to treat the cancers in the phase IV of the Clinical Trials data up to a target year. To create the negative training set, we randomly chose pairs of PKs and cancers where there was no evidence of treating the cancers by inhibiting the PK in the Clinical Trials data up to the target year. The negative training set was chosen to be ten times the size of the positive training set.

### Historical validation: test sets

Independent test sets were chosen from Clinical Trials data subsequent to the target year. The negative test set was chosen to be 10 times larger than the positive test set. No PK-cancer pair was common to both the negative training set and negative test set. In some experiments, the positive test set was defined on the basis of phase I, II, III, and IV studies, i.e. it contained pairs of PKs and cancers where the PKIs were approved to treat the cancers in at least phase I of the Clinical Trials data after the target year (denoted ‘all clinical trial phases’ in Figures 3 and 4). In others, we attempted to predict phase IV trials only (denoted ‘phase IV clinical trials’ in Figure 5). In both cases, as well as in the ‘new’ predictions, we excluded PK/cancer pairs for which there was any trial involving a specific cancer with a PKI that inhibited the PK in the Clinical Trials data in any phase through the end of the target year.

Note that for all predictions, only phase IV data were used for training.

### Random forest learning

The next step after generating positive/negative training/test sets which contain lists of PK/cancer pairs is to find the embeddings of PKs and cancers and prepare the datasets for the prediction task. For a given PK/cancer pair, we subtracted the vector corresponding to the cancer from the vector corresponding to the PK. The difference vectors from the positive training and test sets were labeled with 1 and the difference vectors from the negative training and tests were labeled with 0.

Random forest learning was executed in Python 3.7, using scikit-learn 0.24.1. A randomized search was performed on different parameters including number of estimators, maximum features, maximum depth, minimum samples split, minimum samples leaf and bootstrap using scikit-learn's RandomizedSearchCV function. The best model was selected for the prediction task.

### Concept co-occurrence analysis

As a baseline against which to compare our approach, we implemented a simple classification algorithm that searches the same PubMed abstracts as used above and counts the number of times a concept for a protein kinase is mentioned in the same abstract as the concept for a cancer, classifying the PK-cancer pair as positive if there are at least $k$ co-occurrences, and negative otherwise, for $k\; = \;1,2, \ldots ,25$.

### Performance assessment

The results of predictions are measured by the area under the ROC curves (AUROC). AUROC is a measure of the ability of the classifier to distinguish between the two classes (PK-cancer pairs and non PK-cancer pairs). We additionally assess performance by Precision-Recall (PR) curves, which represent an alternative to ROC curves for tasks with a large skew in the class distribution.

## RESULTS

We developed a machine learning approach that leverages knowledge latent in the published literature to predict pairs of PKs and cancers (henceforth referred to as PK/cancer pairs) that will be the subject of clinical trials registered in the ClinicalTrials.gov resource. Our assumption is that a correct prediction of a future clinical trial of any phase is of interest because it indicates that current scientific knowledge about a PK and a cancer was sufficiently convincing to motivate the investment in a clinical trial. Correct prediction of a future phase IV trial is an indication that inhibition of a PK may be effective in the treatment of a given cancer, because a phase IV trial would be initiated only after a successful phase III trial.

Our pipeline assigns embeddings to words and concepts in the original texts, extracts embeddings related to cancers and PKs, and applies random forest classification to predict pairs of cancer and PKs that correspond to clinical trials in which a PKI that inhibits the PK is used to treat a given form of cancer.

**Figure 1.**
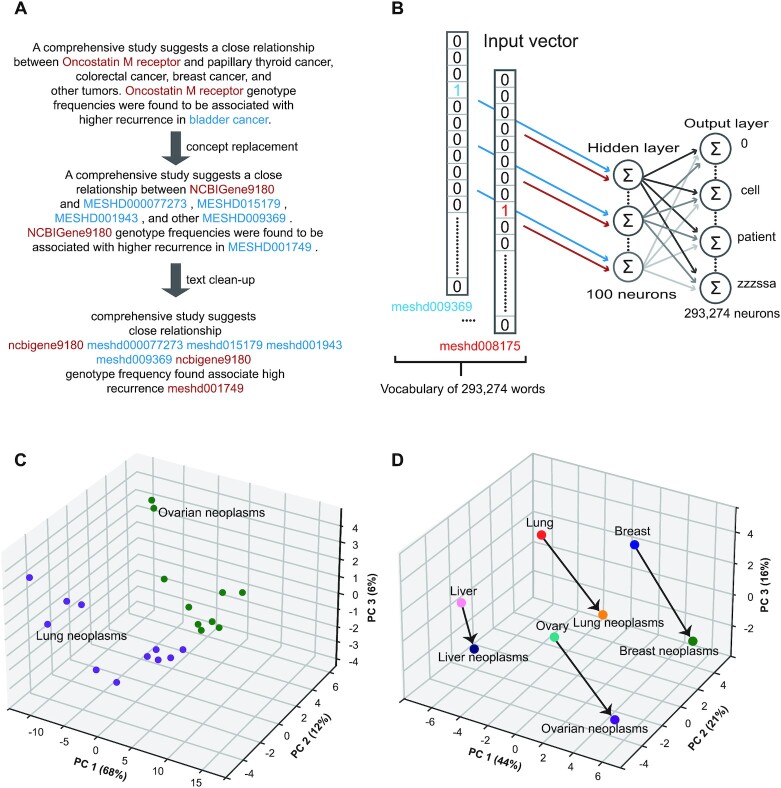
Overview of concept embedding algorithms. (**A**) An example of preprocessing on a text. (**B**) Word2vec skip-gram learning. Words (potentially replaced by concept IDs) are transformed from a one-hot representation into a low-dimensional vector through a one hidden-layer neural network trained to predict context words. Backpropagation learning adjusts the weights of the hidden layer whose output can be interpreted as a low dimensional semantic representation (word vector) of the one-hot encoded input word. The output layer contains probabilities of a word to occur at a neighboring position to the target word. (**C**) Word vectors are represented in the space induced by the first three principal components. Vectors representing Lung Neoplasms and descendent terms are shown in purple; vectors representing Ovarian Neoplasms and descendent terms are shown in green. (**D**) Positions of 8 vectors in three-dimensional PCA space are shown. Arrows are used to connect pairs of vectors representing tissues and cancer that affects the tissue. It can be seen that the pairs form analogies such that, for instance, $f( {Lung} ) - f( {Lung\;neoplasms} ) \approx f( {Breast} ) - f( {Breast\;neoplasms} )$, where *f(.)* represents the embedding of a word in the vector space. Panels (C and D) are meant to illustrate the difference between node classification (panel C) and construction of difference vectors for classification (panel D) but were not used for the actual analysis reported here.

To this end, we selected PubMed articles from 1939 to 2020 (with a gap of 7 years from 1940 to 1946) according to their MeSH descriptors for neoplasms and PKs, obtaining 2 779 507 relevant articles on the basis of 698 MeSH terms for neoplasms and 218 MeSH terms for PKs. We first prepared the abstract texts for word embedding by concept replacement, stop word removal and lemmatization (Figure 1A). The preprocessing step has several desirable effects. First, it merges synonyms; for instance, ‘breast cancer’ and ‘Cancer of Breast’ are both replaced by the corresponding concept id, MESHD001943. Lemmatization replaces inflected word forms with a common base form, for instance ‘higher’ is replaced by ‘high’ in the example of Figure 1A. Stop words, i.e., common words such as ‘a’ and ‘and’, are removed because they do not carry much semantic information. All punctuation marks such as ‘,’ and ‘.’ are removed and all letters are converted to $f( {Lung} ) - f( {Lung\;neoplasms} ) \approx f( {Breast} ) - f( {Breast\;neoplasms} )$ lowercase.

Following this, word embedding was performed with a skip-gram model (Figure 1B). This step creates 100-dimensional vector representations (embeddings) of the words and concepts of the processed abstract texts. The motivating idea of the word2vec algorithm is that because words with similar meanings often appear together, the corresponding embeddings will be located close to each other in the vector space (9). In addition, word vectors may reflect semantic relationships between words in ways that can be expressed as analogies, e.g., France is to Paris as Germany is to Berlin (10). In our data, embeddings for ovarian neoplasms and lung neoplasms formed two distinct clusters (Figure 1C). Additionally, we identified pairs of vectors that demonstrated the semantic relation ‘organ-specific cancer relates to organ’ (Figure 1D).

### PKIs targeting PKs

The goal of our approach is to predict clinical studies related to therapeutically relevant PK-cancer pairs. To do so, we curated information available in DrugCentral (19) and identified 75 PKIs that have been used to treat cancers. In many cases, the PKIs inhibit multiple PKs at a <0.3 μM cutoff, and a total of 84 PKs are inhibited by these kinases. The mean number of PKs inhibited by a given PKI was 2.8 (median 2, min. 1, max. 5), and the mean number of PKIs that inhibit a given PK was 2.5 (median 2, min. 1, max. 13) (Supplementary Material Figure S1A and S1B). We retrieved clinical studies that involved these PKIs from the ClinicalTrials.gov resource (20), identifying 2105 phase I, 3185 phase II, 555 phase III, and 217 phase IV studies performed between 1991 and 2021 (total 6062; Supplementary Material Figure S2A and S2B).

### Random forest classification of PK-cancer pairs

**Figure 2.**
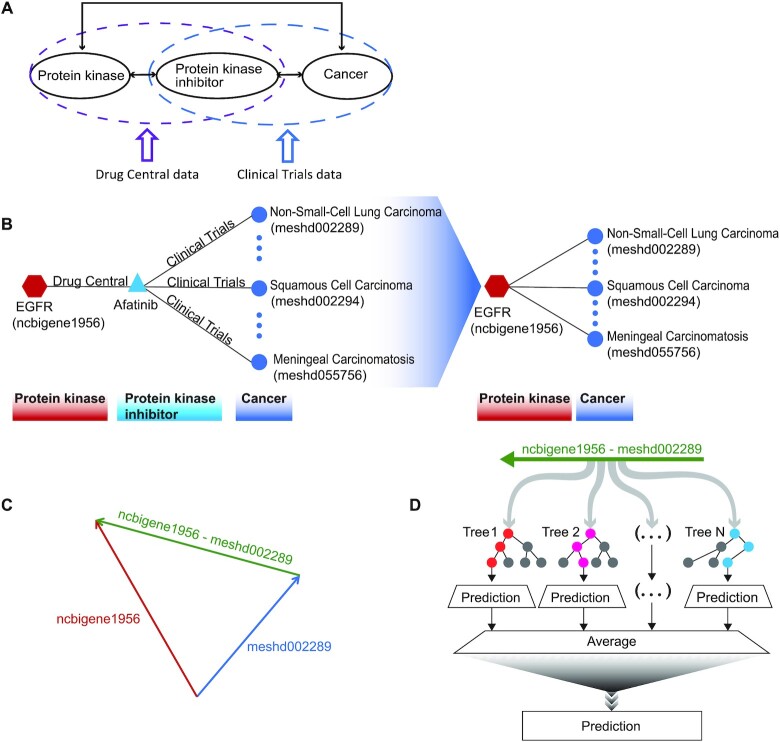
Machine learning to predict PKs relevant for treating cancer. (**A**) Information about Phase I-IV trials of PKIs was retrieved from ClinicalTrials.gov and DrugCentral. (**B**) A simplified example showing how the PK-cancer pairs were derived from ClinicalTrials.gov and DrugCentral. (**C**) The embedded vectors derived from skip-gram analysis of PubMed abstracts were used to generate ‘analogy vectors’ by subtracting vectors of cancers from vectors of PKs. Positives were defined by the Clinical Trials data, and negatives were chosen from the remaining vectors. (**D**) Random forest classification was applied with analogy vectors as input.

We then used the word embeddings as the basis for machine learning classification. We first extracted the 698 embeddings representing neoplasms and the 218 embeddings for PKs. For the 75 PKIs that have been used to treat cancers, we extracted information from DrugCentral regarding the PKs that are inhibited by each PKI with the highest affinities (see Materials and Methods for details). We then extracted data from ClinicalTrials.gov about clinical trials in which the use of the PKI to treat a certain cancer was investigated. We interpret a phase IV (postmarketing) trial as evidence that the PKI demonstrated efficacy in treating the cancer. Figure 2B offers an example of how our procedure would associate EGFR with three cancers against which the PKI afatinib demonstrated efficacy.

It can be seen from Figure 1D that only some pairs of tissues and cancers form valid analogies. For instance, $f( {Lung} ) - f( {Lung\;neoplasms} ) \approx f( {Breast} ) - f( {Breast\;neoplasms} ),$ while it is not true that $f( {Lung} ) - f( {Breast\;neoplasms} ) \approx f( {Breast} ) - f( {Lung\;neoplasms} )$. We reasoned that vectors of the form $f( {PK} )\; - \;f( {Cancer} )$ could be used for classification if the distribution of vectors derived from PKs whose inhibition can be exploited to treat a given cancer differs from the general distribution of vectors derived from arbitrary pairs of PKs and cancers. For instance, the PKI sorafenib inhibits the kinases RAF, BRAF, FLT3, VEGFR 1–3, PDGFR, c-KIT and RET and significantly improves progression-free survival compared with placebo in patients with progressive radioactive iodine-refractory differentiated thyroid cancer (21). For the purposes of our analysis, the positive set includes vectors formed by subtracting the vector for Thyroid Neoplasms (MeSH D013964) from those for the above-mentioned nine PKs. We assume that the vast majority of relations between PKs and cancers are not therapeutically relevant in this way, although data to prove this negative role is not generally available in the literature. On this assumption, vectors that are not in our positive set are considered negative.

It is worth noting that several relations between words, including analogy, are approximately preserved by simple linear combinations (e.g. subtraction) of the vectors representing the words in the embedding space (22). Here, for each PK-cancer pair, we define a difference vector by subtracting the cancer vector from the corresponding PK vector (Figure 2C). The sets of positive and negative vectors defined in this way are used for random forest learning. The features used by the random forest are provided by the values of each of the 100 dimensions of the embedded vectors (Figure 2D).

**Figure 3.**
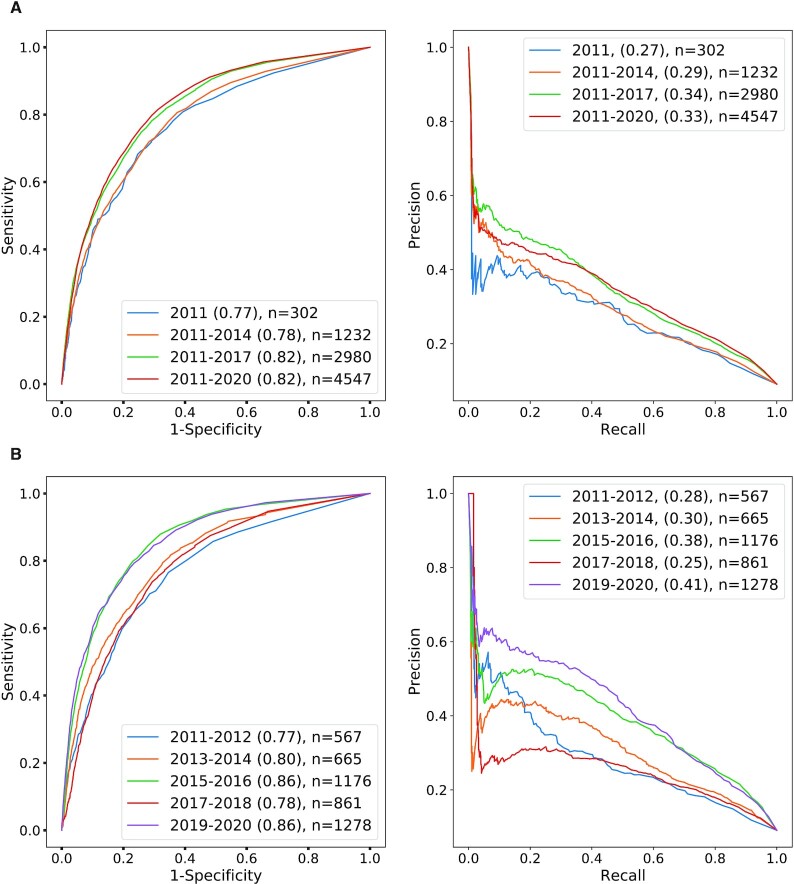
ROC/PR analysis of predicted PK-cancer pairs (2010, all clinical trial phases). ROC and PR curves for predictions based on abstracts published up to 2010. Test data are PK/Cancer pairs derived from clinical trials and DrugCentral PK/PKI information as described in Materials and Methods. (**A**) Tests are arranged in 1-, 4-, 7-, and 10-year periods starting from 2011. (**B**) Tests are arranged in non-overlapping 2-year periods starting from 2011. See Supplementary Table S2 for the threshold that achieves the optimal F1 score with the precision and recall values at that threshold. In both panels, the number of PK-cancer pairs in positive test sets is shown with *n*.

**Figure 4.**
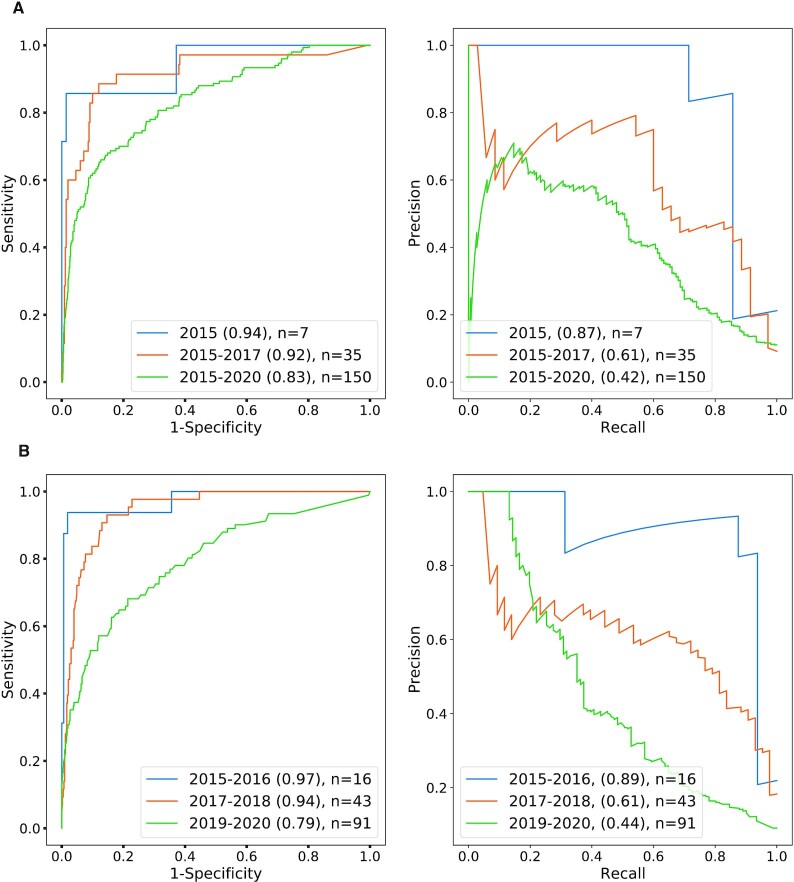
ROC/PR analysis of predicted PK-cancer pairs (2014, all clinical trial phases). ROC and PR curves for predictions based on abstracts published up to 2014. Test data are PK/Cancer pairs derived from clinical trials and DrugCentral PK/PKI information as described in the methods. (**A**) Tests are arranged in 1-, 3-, and 6-year periods starting from 2015. (**B**) Tests are arranged in non-overlapping 2-year periods starting from 2015. See Supplementary Table S4 for the threshold that achieves the optimal F1 score with the precision and recall values at that threshold. In both panels, the number of PK-cancer pairs in positive test sets is shown with *n*.

As an example of our procedure, we describe the historical validation pipeline for the target year of 2010 in detail. About 2533 clinical trials were registered in ClinicalTrials.gov between 1991 and 2010, resulting in 107 PK-cancer pairs. The negative training set was constructed by randomly choosing 1070 PK-cancer pairs not mentioned in the ClinicalTrials.gov data in 2010 or before (see Materials and Methods for more details). Random forest classification was trained on the difference vectors obtained by subtracting vectors corresponding to cancers from vectors corresponding to PKs in the training set. The parameters of the random forest classifier are explained in Materials and Methods. In our first analysis on historical predictions, we evaluated the classification performance on a test set of newly recorded clinical trials in ClinicalTrials.gov for 2011 (1 year after 2010), 2011–2012 (2 years after 2010), 2011–2014 (4 years after 2010) and so on up to 2011–2020 (10 years after 2010). The number of positive test examples is shown in the figures, and ten times as many negative examples were chosen as described above. The AUROC scores start from 77% in 2011, immediately one year after 2010 and stay within the same range between 78% and 82% over the following time periods, reaching the AUROC score of 82% for 2011–2020; the average precision ranged from 27% to 34% (Figure 3A). In our second analysis, we evaluated the classification performance on a test set of newly recorded clinical trials in 2011–2012, 2013–2014, and so on up to 2019–2020. The AUROC was 77% for data in the first 2 years immediately following the target year, showed some fluctuations in the next 2-year intervals, and reached around 86% in 2015–2016 and 2019–2020. The AUROC ranged from 77% to 86%, and the average precision ranged from 28% to 41% (Figure 3B). We performed an analogous analysis with a target year of 2014 (Figure 4).

We then attempted to predict the appearance of phase IV clinical trial studies for PK-cancer pairs with an experimental approach that was otherwise identical to the above. There are many fewer positive test examples when limiting the data to phase IV. For the 10-year period 2011–2020, there were 295 positive test examples; the AUROC was 87% and the average precision was 43% (Figure 5 and Supplementary Figure S14). Results for other values of the PK-per-PKI parameter are shown in Supplementary Figures S3–S15.

**Figure 5.**
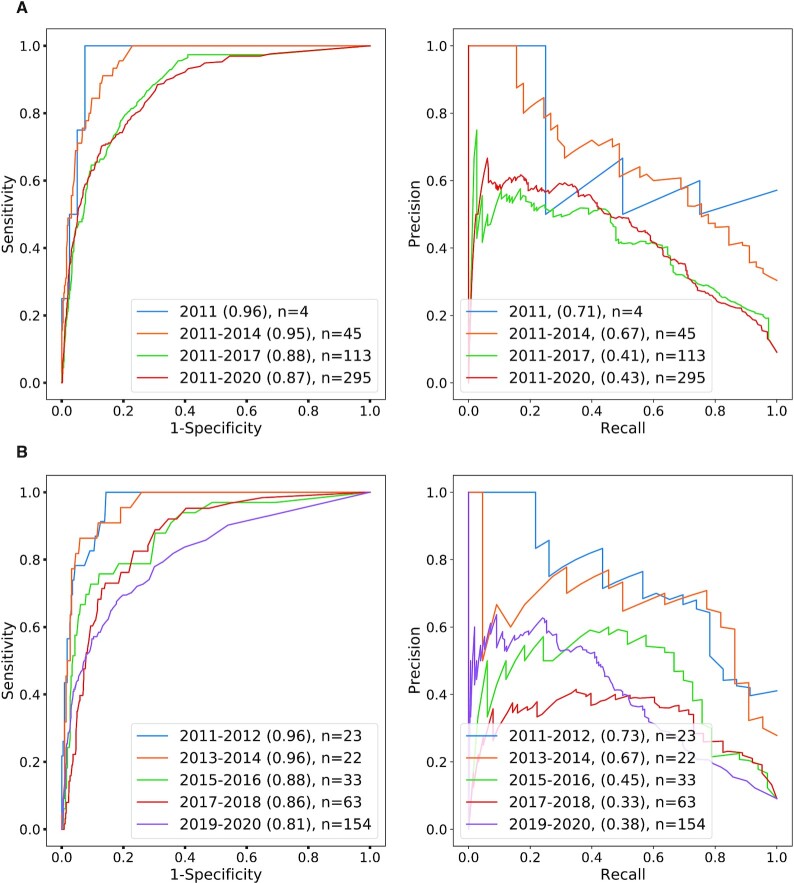
ROC/PR analysis of predicted PK-cancer pairs (2010, phase IV clinical trials). ROC and PR curves for predictions based on abstracts published up to 2010 with test data limited to phase IV trials. Groups and explanations are analogous to those in Figure 3.

In the manuscript, a PK-per-PKI threshold (Materials and Methods) of 5 is shown. Supplementary Figures S3–S15 show ROC and precision-recall curves for phase IV and all phases for PK-per-PKI thresholds of 1, 2, 5, 10 for results not shown in the main manuscript, and Supplementary Tables S1–S6 present a summary of results from all experiments. There was no value of the PK-per-PKI threshold that maximized the AUROC or area under the PR curve for all experiments.

In order to assess the additional value of our approach above a simple co-occurrence analysis (Materials and Methods), we analyzed the performance of predicting valid cancer-PK pairs based on a threshold number of abstracts in which both concepts are mentioned, varying the threshold from 1 to 25. The maximum F1 score was 0.242 for predicting all phases, and 0.087 for predicting phase IV studies (Supplementary Tables S7 and S8).

Finally, we ran our method on the entire corpus of PubMed abstracts up to November 2020. We considered all clinical trials through 2020 and also clinical trials that have been verified in 2021. We then constructed the positive training set using all PK-cancer pairs from clinical trials of phase IV. The negative training set contains randomly generated pairs of PKs and cancers where there was no evidence in the clinical trials data of treating the cancer by inhibiting the PK. Similar to the historical prediction analysis, we chose the size of the negative training set to be 10 times the size of the positive training set. The prediction set includes all possible PK-cancer pairs except those where there was evidence of inhibiting the PKs in any of phase I, II, III or IV clinical trials that have been registered so far. The prediction set also contains PK-cancer pairs for PKs that have not been targeted yet. The size of the positive training set, negative training set and prediction set are 557, 5570 and 325 494 examples, respectively.

In Supplementary Material File S3, we have provided the predictions with prediction scores at least 0.491. This value was chosen based on the threshold of the AUROC scores which maximizes the geometric mean of the sensitivity (True Positive Rate, TPR) and specificity (1 – False Positive Rate, FPR), i.e., sqrt(TPR * (1-FPR)). 2979/325494 (0.92%) of the predictions were above this threshold. The predictions include many that flag an additional indication for inhibition of a kinase that is targeted by PKIs in existing trials. For instance, the second most highly ranked prediction is for KDR and hepatocellular carcinoma. KDR was shown to be a regulator of vascular endothelial growth factor–induced tumor development and angiogenesis in murine hepatocellular carcinoma cells (23). Similarly, the twelfth prediction is for CSF1R and giant cell tumors. This is of potential interest since tenosynovial giant cell tumors (TGCTs) are characterized by rearrangements of CSF1, which is a ligand for CSF1R (24). According to the prediction at rank 37, the PK *RYK*, which according to our Drug Central data has not been targeted yet, was found to be a potential target in *lung neoplasms*.

## DISCUSSION

*De novo* drug development typically costs several billion U.S. dollars, takes 13–15 years, and suffers a high failure rate (25–27). Phase I trials are typically performed after pre-clinical studies have suggested the potential utility of an investigational medication for a certain disease. However, <10% of medications entering phase I clinical testing will achieve FDA approval and reach the market (28,29). This has motivated the development of computational methods to reduce risk and increase efficiency of novel drug development. Myriad computer-aided drug discovery/design methods have been developed with a number of different approaches (30). High-throughput screening (HTS) is a brute force method that investigates high numbers of molecules to find those that elicit a desired response. Virtual screening is a strategy that prioritizes compounds computationally so that HTS experiments can concentrate on subsets of compounds most likely to have the desired activity. The high degree of structural homology among protein kinases makes poorly studied kinases interesting targets for homology modeling and virtual screening (31). Numerous computational approaches have been published (32), including a Kinase Atlas to explore allosteric sites in kinases (33).

Drug repurposing aims to find novel targets and clinical uses for already known drugs (34). A broad range of computational methods have been developed, many of which construct networks (graphs) that comprise information about features such as mechanism of action, chemical and physiological processes, diseases, drugs, gene expression and others (27,35–37). Drug repurposing is an attractive strategy for PKIs, and a number of PKIs originally developed for one indication have been successfully repurposed for others (38). However, one major challenge is that although many PKIs inhibit multiple kinases, the complete bioactivity matrix (PKIs versus kinases) remains poorly characterized (5). Computational approaches to repurposing PKIs for cancer have leveraged gene expression profiles (39–41), systems biology (42), and deep learning (43).

In this work, we investigate whether the inhibition of a specific protein kinase (rather than use of a specific protein kinase inhibitor) could be associated with a beneficial response for a certain cancer. We do so by linking PKIs to the protein kinases they inhibit and then linking PKIs to specific cancers based on information in ClinicalTrials.gov. Our work leverages word2vec (10) to generate embeddings of concepts across a large subset of abstracts in the PubMed resource as a foundation for machine learning. The key concept of word2vec goes back to the dictum of John Firth from 1957: ‘You shall know a word by the company it keeps’ (44), meaning that context words that tend to appear near a target word in a text corpus encode information about the word's meaning. The embedding vectors can be regarded as a compact representation of the meaning of the words in a vector space. Semantically related words tend to be close to each other in the vector space. Additionally, the relative positions of pairs of words reflects the relation between them (10). For instance, if $f$ is the mapping from a large text corpus to a vector space, we often find the vectors encode similarities that capture the gender relation, $f( {woman} ) - f( {queen} ) \approx f( {man} ) - f( {king} )$, or the language-spoken-in relation, $f( {Germany} ) - f( {German} ) \approx f( {Italy} ) - f( {Italian} )\;$(45).

The basic idea of our algorithm is that an embedding can capture the relations between entities of two different sets, but only some potential relations are true. For instance, the relation country–capital city is a mapping from the set of countries to the set of capital cities. The relations *France–Paris* and *Italy–Rome* are true, but the relation *France–Rome* is false.

In the biomedical sciences, there are myriad relations where we know of a limited number of true relations but are striving to identify the complete set of true relations. For instance, inhibition of PK activity has proved to be an effective anti-cancer treatment, but it is not true that inhibiting an arbitrary PK is an effective treatment for an arbitrary cancer. Only a subset of all potential pairs of PKs and cancers are true in the sense that inhibiting the PK will effectively treat the cancer. If we could accurately predict such pairs, then one could focus efforts on clinical trials for PKIs that inhibit the most relevant PK-cancer pairs.

Word embedding methods can represent an entire vocabulary of words in a relatively low-dimensional vector space, where semantic similarities between words are preserved in the corresponding embedded linear space (10). The embedded vectors generated by word2vec can be used as input for classification algorithms (46–48). Vector cosine similarity in an unsupervised word embedding enabled the prediction of applications for materials years before their publication in the materials science literature (49). Several supervised analogy learning methods based on word embeddings have been successfully applied in a variety of natural language processing tasks (22,50,51). Our algorithm uses this approach to leverage information about cancer and kinases latent in the published literature.

Our methodology could be extended to other biomedical research questions that can be framed as a search for valid relations between concepts from two different sets. The word2vec step could be replaced by more advanced word embedding methods such as Bidirectional Encoder Representations from Transformers (BERT) (52), including the SciBERT version trained on the scientific literature (53). The concept replacement step could be extended to encompass additional terminologies or concept recognition algorithms. To classify difference vectors, we could replace Random Forest with many other classification algorithms.

### Limitations

The algorithm presented here aims to identify PK/cancer pairs with potential therapeutic relevance: inhibition of the kinase can have beneficial effects in treating the cancer. We are not attempting to predict the suitability of PKIs for individual patients, which may be complicated by many factors such as genetic variability and the acquisition of resistance to a particular targeted treatment. This is beyond the scope of our method.

All phase IV studies come after FDA approval, but not all FDA-approved drugs undergo phase IV studies. Our predictions may be conservative. ClinicalTrials.gov tracks <50% of clinical studies worldwide, and thus our training data is incomplete. We know of no standardized database that has the current status of all PKIs with the results of clinical trials for the cancers they have been used to treat. Clinical trials may return negative results for many reasons, including a high incidence of side effects, better performance by a competing drug, or inability to recruit sufficient patients for the trial. Even if we had an accurate and comprehensive database of negative results for clinical trials, we could not use this information to infer reliably that there is no therapeutic relation between a protein kinase and a certain cancer type. Therefore, the negative examples used in this work were chosen from the set of all possible combinations of protein kinases and cancers, under the assumption that the majority of these are not therapeutically valid.

## CONCLUSION

This work presents a novel approach to predict new associations between PKs and cancers, meaning that by targeting the PKs, the corresponding cancers could be treated. We first used a word embedding algorithm to map words of PubMed abstracts to vectors. We then applied a Random Forest classifier to predict new PK/cancer pairs after training on the embedded vectors of known PK/cancer pairs obtained from Clinical Trials and Drug Central data. We assessed our method with historical prediction and obtained an average AUROC above 0.8. We deployed our method on the entire corpus of PubMed abstracts and all known PK/cancer pairs currently available, to predict novel PK/cancer pairs. We found new associations between certain types of cancer and PKs that have not yet been targeted.

The main methodological innovation of our work is our approach to the discovery of latent knowledge about the relationship between concepts from two different categories. Previous work has shown that concept embedding in material science literature followed by machine learning classification can recommend materials for functional applications several years before their discovery (49). This approach represents binary classification of individual embedded concept vectors. In contrast, our approach investigates two classes of concepts (PKs and cancers); existing evidence suggests that only a subset of PK/cancer pairs participate in a ‘therapeutically relevant’ relation (c.f. Figure 2C), whereby inhibition of a specific protein kinase contributes to the treatment of a certain cancer. Our approach attempts to identify such therapeutically relevant relations between concepts prior to their publication in the medical literature. There are numerous other areas in which interesting classification tasks involve the relationships between members of different concept sets that would be amenable to our approach.

## DATA AVAILABILITY

Several code repositories were developed for this project. *marea* performs concept replacement and preprocessing of PubMed abstracts and is available at https://github.com/TheJacksonLaboratory/marea under the BSD 3 license. *Yet another clinical trials parser* (YACTP) retrieves and processes information from ClinicalTrials.gov and is available at https://github.com/monarch-initiative/yactp under the GNU General Public License v3.0. *Kinase Cancer Embedding Tool* (KCET) is available at https://github.com/TheJacksonLaboratory/KCET and contains scripts and Jupyter notebooks used to perform word embedding and to leverage the embeddings for random forest classification. The analysis described in this manuscript corresponds to release v0.4.0. The embedding software, *embiggen*, performs word embedding and is available at https://github.com/monarch-initiative/embiggen as well as via PyPi at https://pypi.org/project/embiggen/.

The repository https://zenodo.org/record/5516252 contains the file that was output from YACTP, representing ClinicalTrials.gov entries for the protein-kinases investigated in this work, as well as files with embeddings and labels from relevant PubMed abstracts up to 2010, 2014 and 2020. These files can be used to run scripts and notebooks in the KCET repository.

## Supplementary Material

lqab113_Supplemental_Files

## Notes

Present address: Vida Ravanmehr, The University of Texas MD Anderson Cancer Center, 77030 Houston TX, USA.
