# Supplementary material for: Supervised learning with word embeddings derived from PubMed captures latent knowledge about protein kinases and cancer
Source: NAR Genom Bioinform. 2021 Dec 8;3(4):lqab113. doi: 10.1093/nargab/lqab113 (PMC8652379; doi:10.1093/nargab/lqab113)
Supplement: lqab113_Supplemental_Files [file lqab113_supplemental_files.zip › List of Supplementary Files.docx]

**Supplementary File 1**. Excel file with protein kinase inhibitors, targets, and affinities, derived from DrugCentral.

**Supplementary File 2**. Excel file with clinical trials data used for this study.

**Supplementary File 3**. Excel file with above threshold predictions based on PubMed data up to 2020.

**Supplementary Material**. Supplemental Figures S1-S16, Tables S1-S8
