# Supplementary material for: Supervised learning with word embeddings derived from PubMed captures latent knowledge about protein kinases and cancer
Source: NAR Genom Bioinform. 2021 Dec 8;3(4):lqab113. doi: 10.1093/nargab/lqab113 (PMC8652379; doi:10.1093/nargab/lqab113)
Supplement: lqab113_Supplemental_Files [file lqab113_supplemental_files.zip › supplement.pdf]

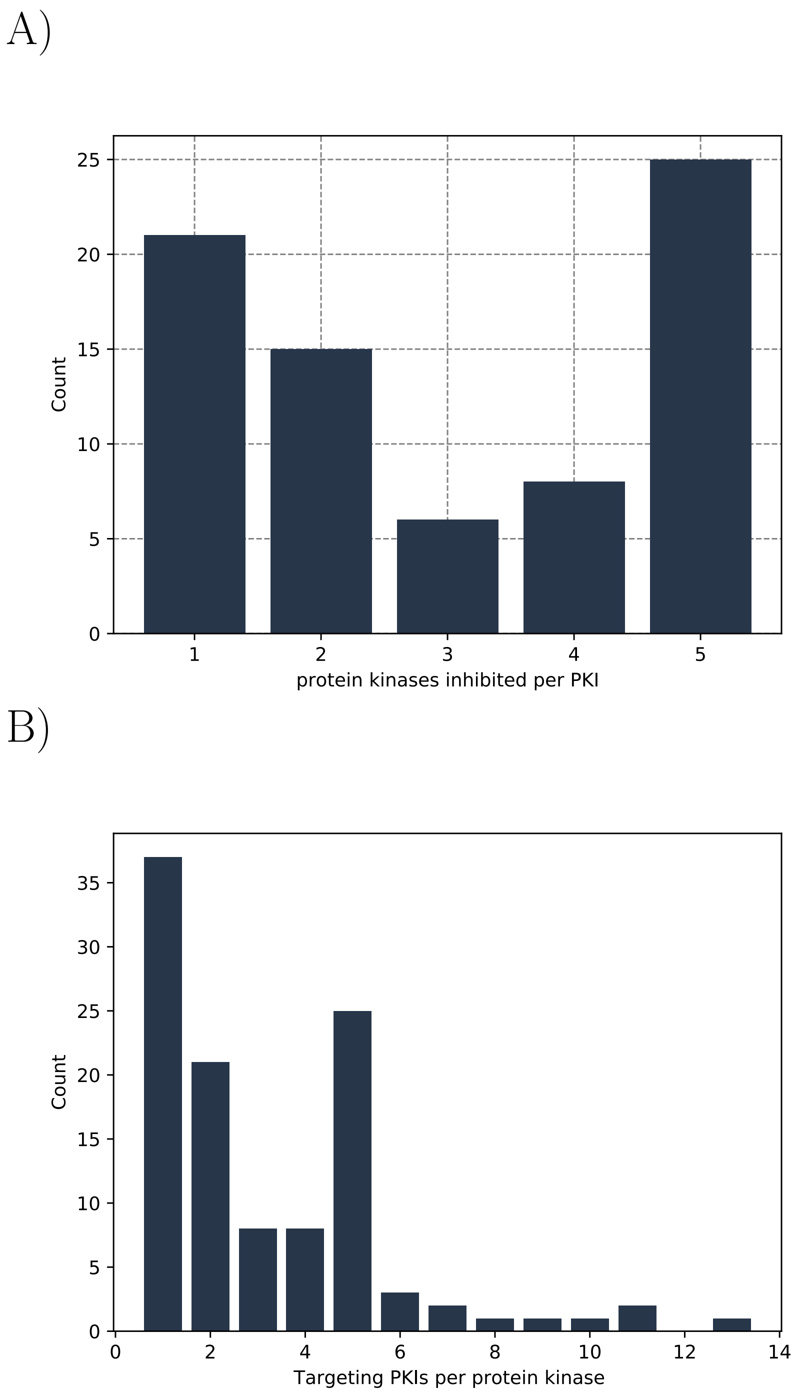

Figure S1. Protein kinases (PKs) and Protein Kinase Inhibitors (PKIs). **A)** The histogram of the number of PKs that are inhibited by a given PKI, **B)** The histogram of the number of PKIs that inhibit a given PK.

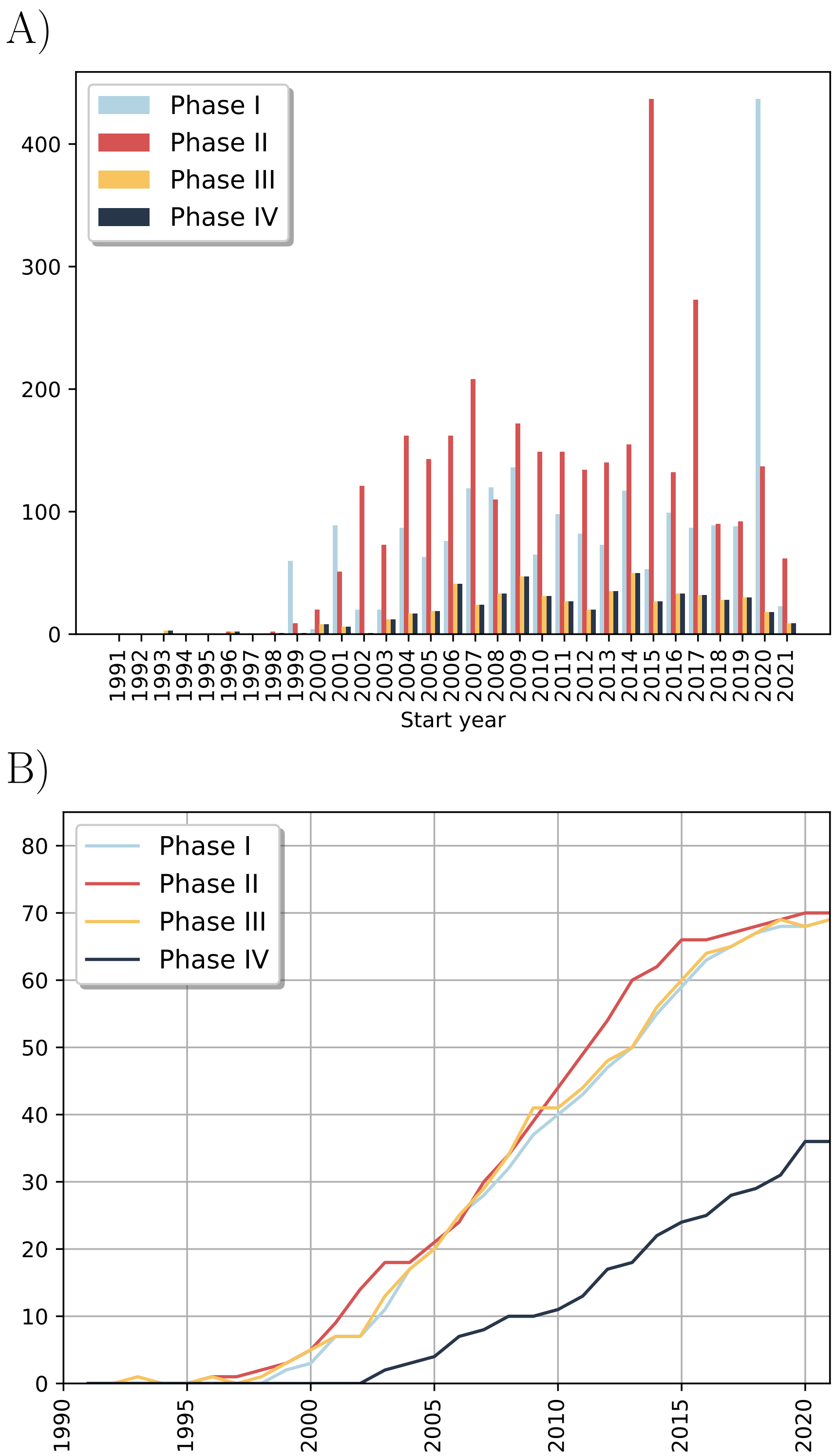

Figure S2. Clinical trials and protein-kinase inhibitors (PKIs) by year. **A)** Histogram of phases I, II, III and IV of the clinical trials data from 1991 to 2021. **B)** Number of PKIs being studied per year and per phase from 1991 to 2021.

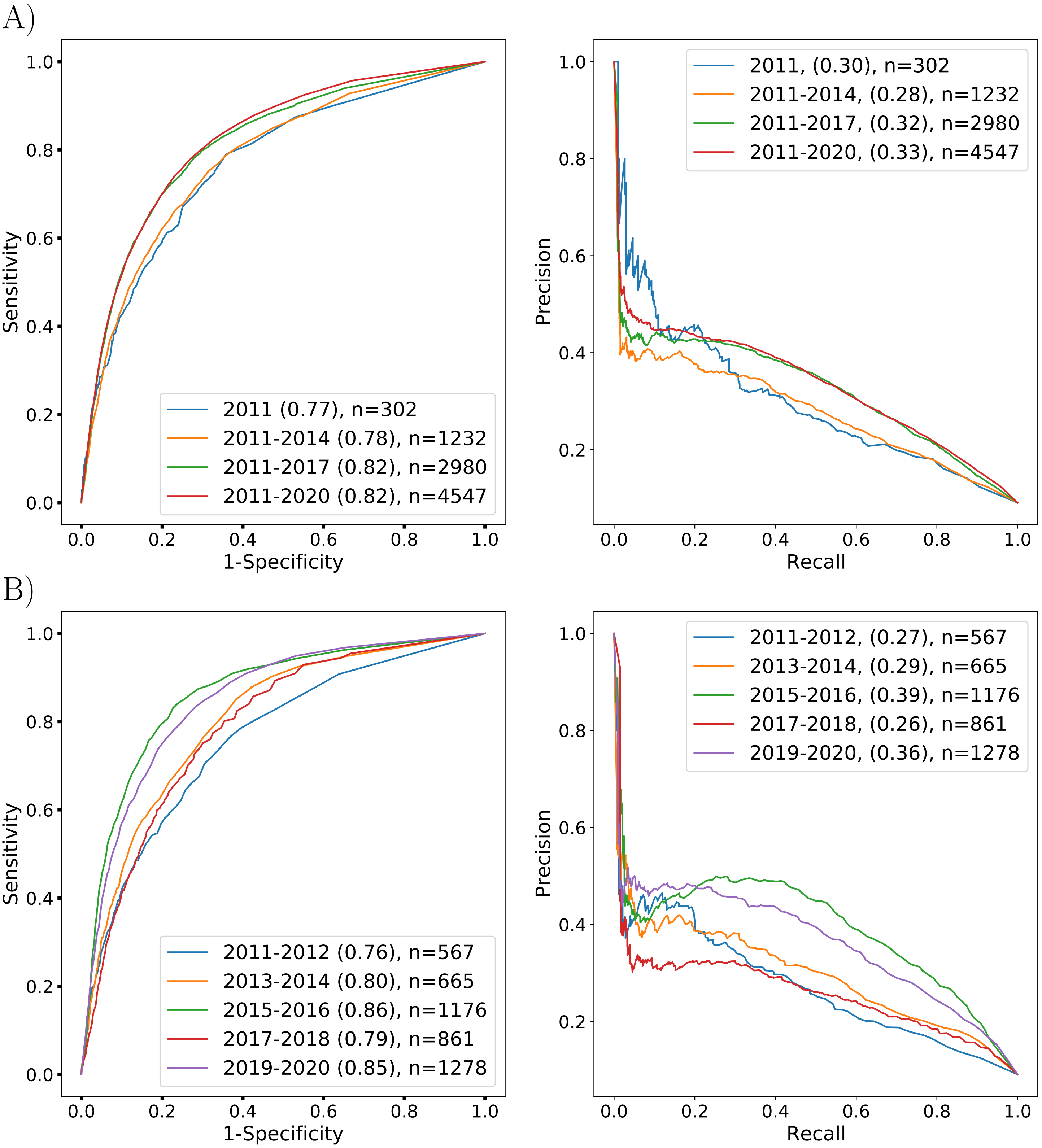

Figure S3. Clinical trials and protein-kinase inhibitors (all phases, 2010, p_ki=1). The left side of each panel shows receiver-operating characteristic analysis (ROC) and the right side shows precision-recall analysis (PR). Training data included abstracts and studies up to 2010. Data from all four phases were included with p_ki=1 (maximum PKs per PKI; see methods). Panels A and B show results with testing data from various periods.

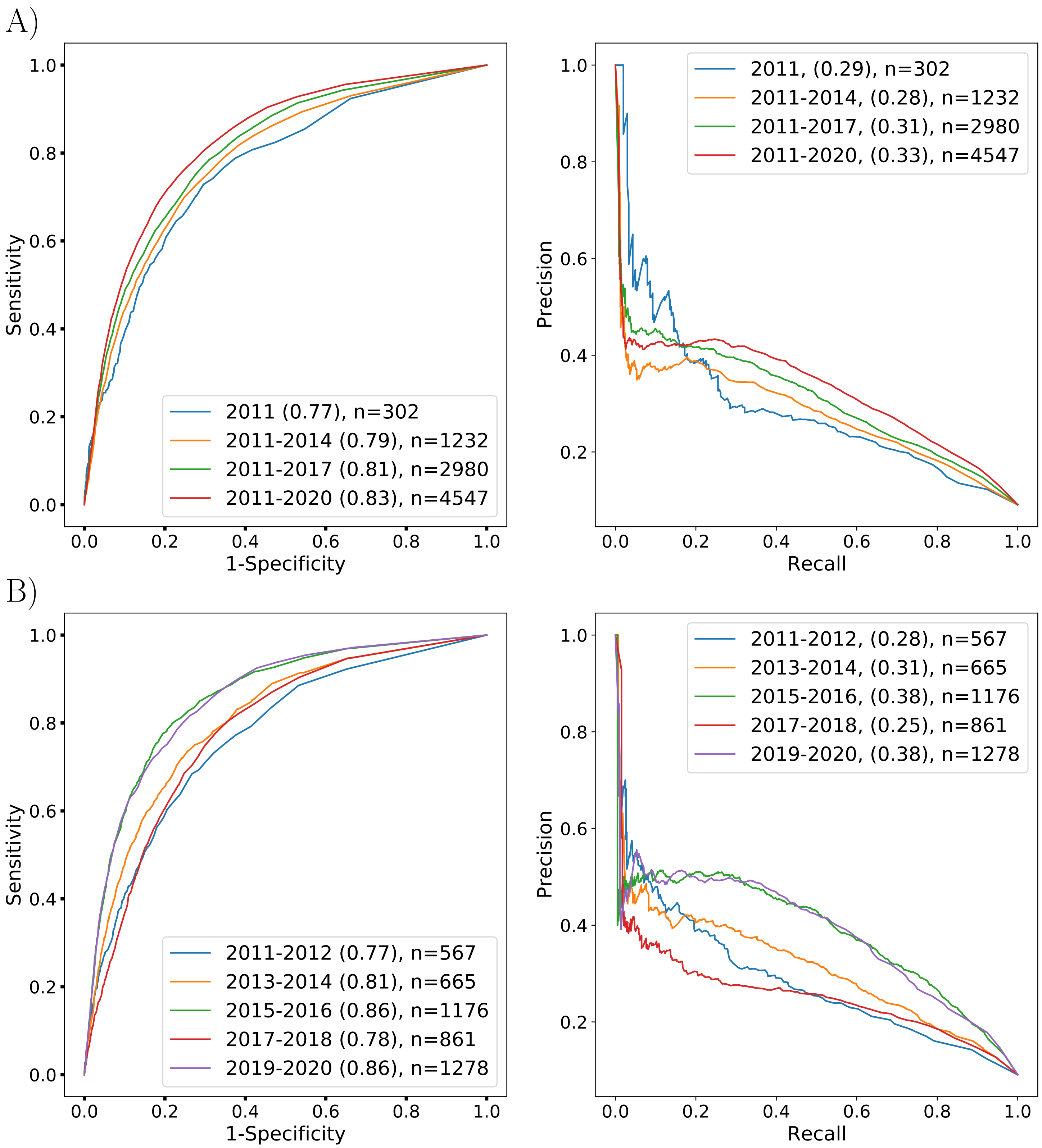

Figure S4. Clinical trials and protein-kinase inhibitors (all phases,2010, p_ki=2). The left side of each panel shows receiver-operating characteristic analysis (ROC) and the right side shows precision-recall analysis (PR). Training data included abstracts and studies up to 2010. Data from all four phases were included with p_ki=2 (maximum PKs per PKI; see methods). Panels A and B show results with testing data from various periods.

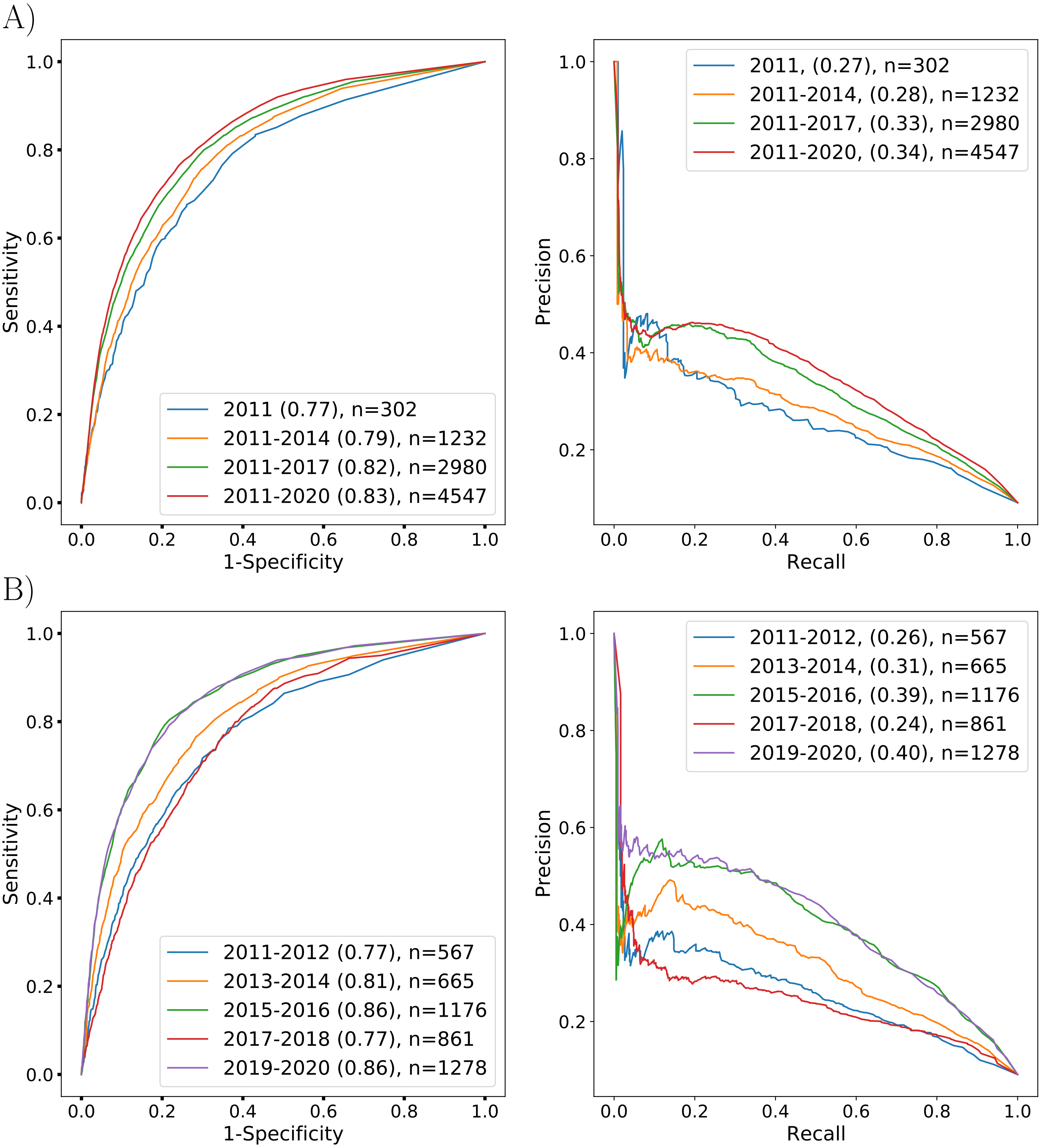

Figure S5. Clinical trials and protein-kinase inhibitors (all phases, 2010, p_ki=10). The left side of each panel shows receiver-operating characteristic analysis (ROC) and the right side shows precision-recall analysis (PR). Training data included abstracts and studies up to 2010. Data from all four phases were included with p_ki=10 (maximum PKs per PKI; see methods). Panels A and B show results with testing data from various periods.

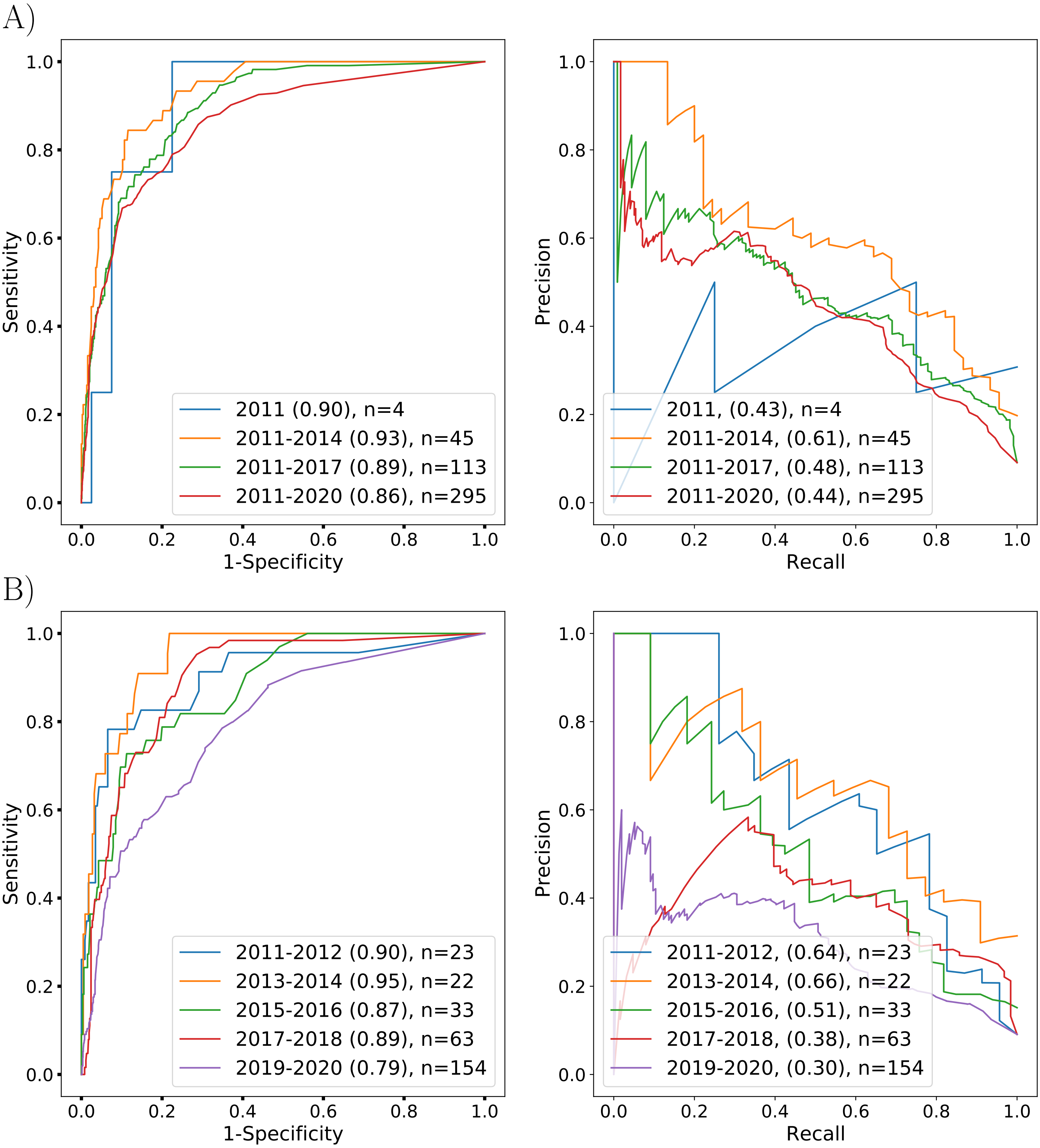

Figure S6. Clinical trials and protein-kinase inhibitors (phase 4, 2010, p_ki=1). The left side of each panel shows receiver-operating characteristic analysis (ROC) and the right side shows precision-recall analysis (PR). Training data included abstracts and studies up to 2010. Data from all four phases were included with p_ki=1 (maximum PKs per PKI; see methods). Panels A and B show results with testing data from various periods.

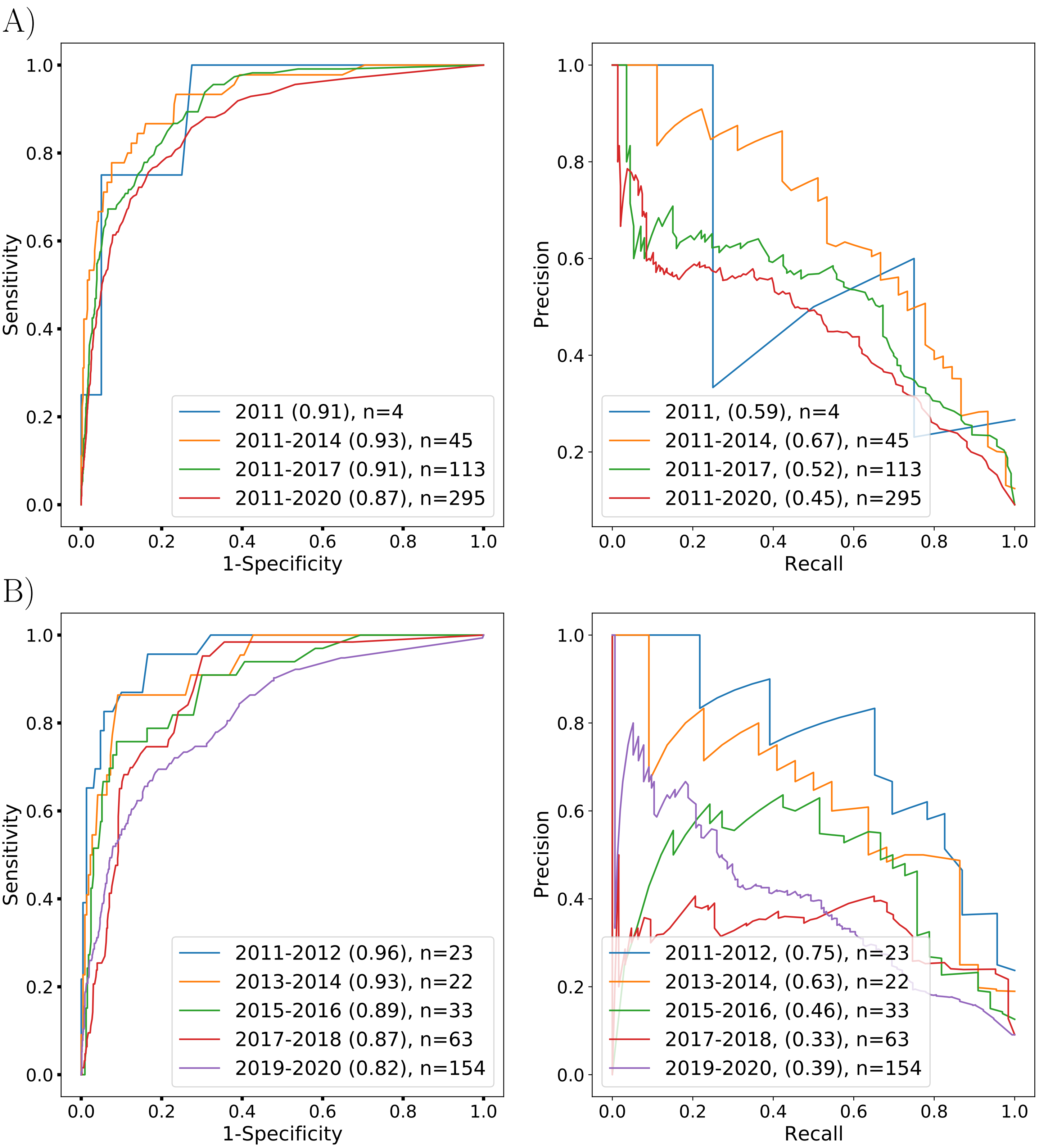

Figure S7. Clinical trials and protein-kinase inhibitors (phase 4, 2010, p_ki=2). The left side of each panel shows receiver-operating characteristic analysis (ROC) and the right side shows precision-recall analysis (PR). Training data included abstracts and studies up to 2010. Data from all four phases were included with p_ki=2 (maximum PKs per PKI; see methods). Panels A and B show results with testing data from various periods.

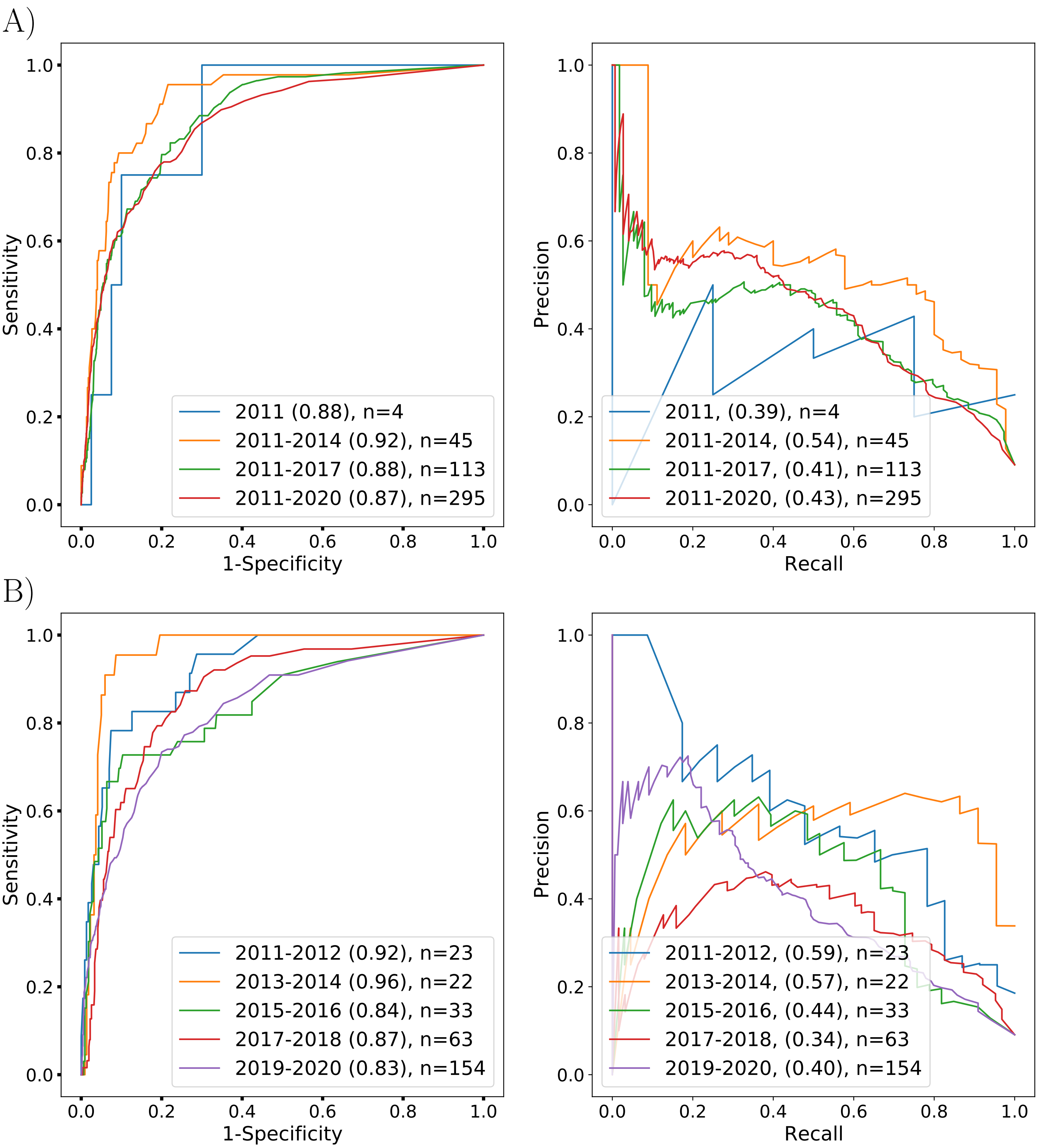

Figure S8. Clinical trials and protein-kinase inhibitors (phase 4, 2010, p_ki=10). The left side of each panel shows receiver-operating characteristic analysis (ROC) and the right side shows precision-recall analysis (PR). Training data included abstracts and studies up to 2010. Data from all four phases were included with p_ki=10 (maximum PKs per PKI; see methods). Panels A and B show results with testing data from various periods.

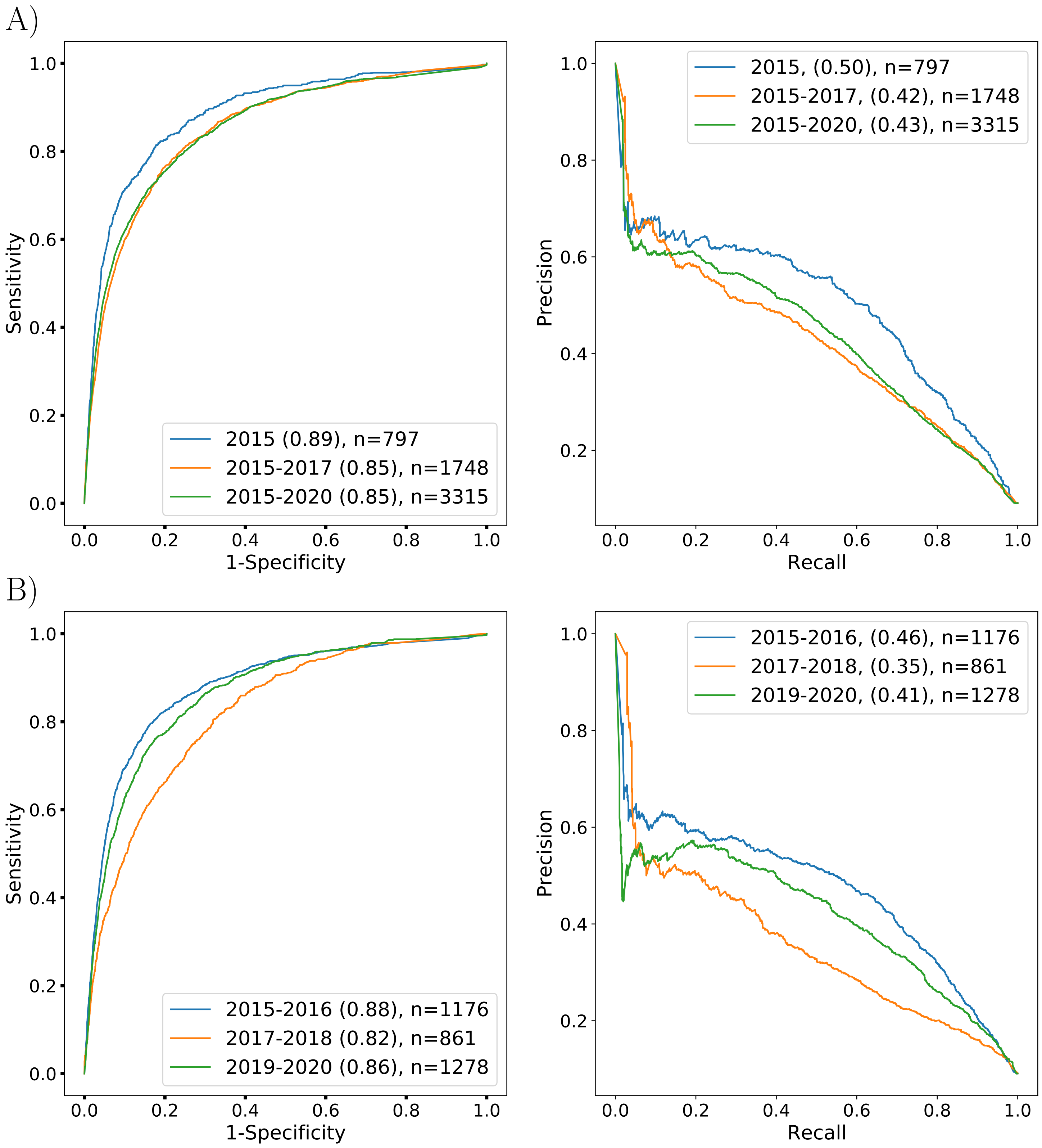

Figure S9. Clinical trials and protein-kinase inhibitors (all phases, 2014, p_ki=1). The left side of each panel shows receiver-operating characteristic analysis (ROC) and the right side shows precision-recall analysis (PR). Training data included abstracts and studies up to 2014. Data from all four phases were included with p_ki=1 (maximum PKs per PKI; see methods). Panels A and B show results with testing data from various periods.

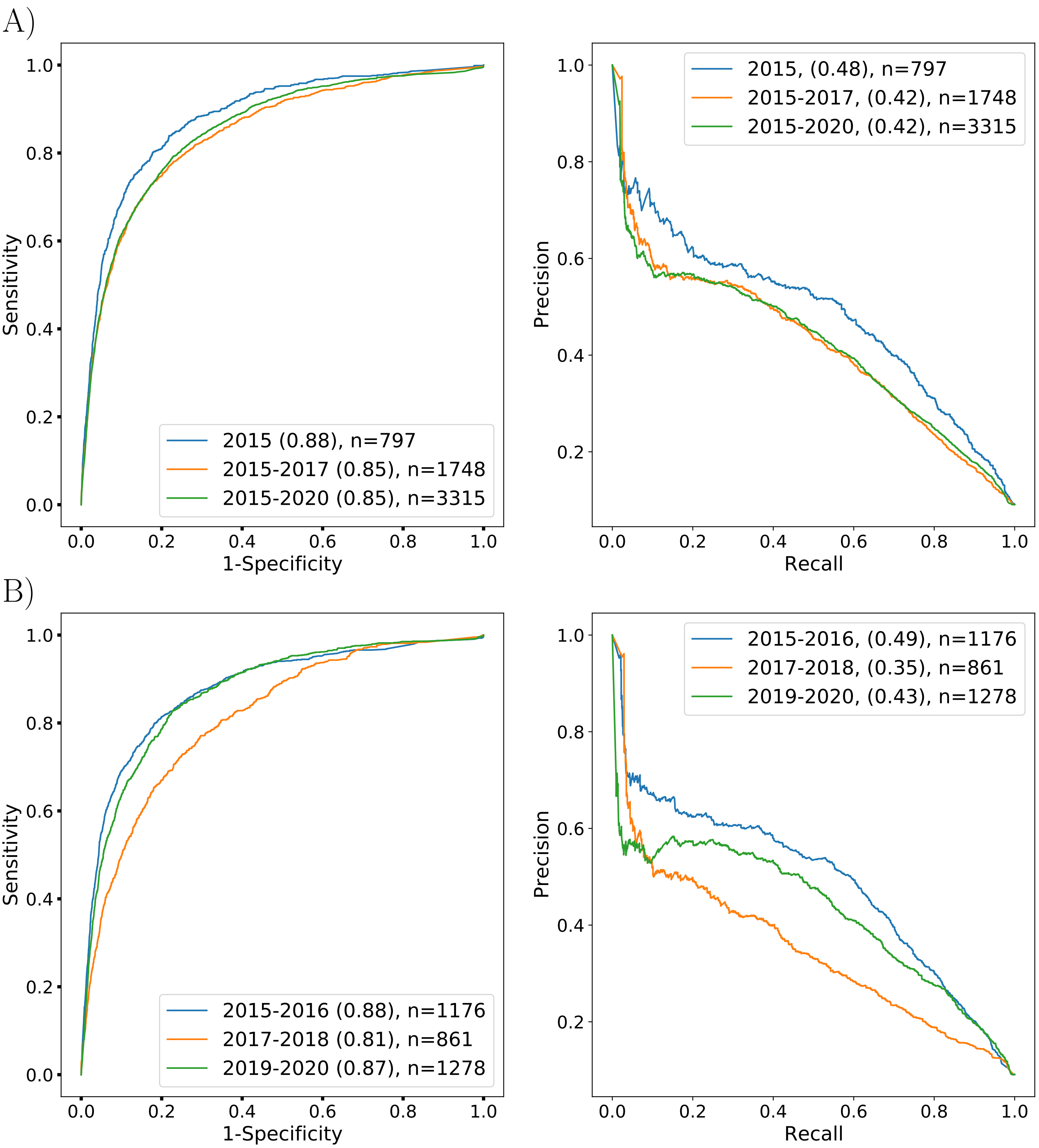

Figure S10. Clinical trials and protein-kinase inhibitors (all phases, 2014, p_ki=2). The left side of each panel shows receiver-operating characteristic analysis (ROC) and the right side shows precision-recall analysis (PR). Training data included abstracts and studies up to 2014. Data from all four phases were included with p_ki=2 (maximum PKs per PKI; see methods). Panels A and B show results with testing data from various periods.

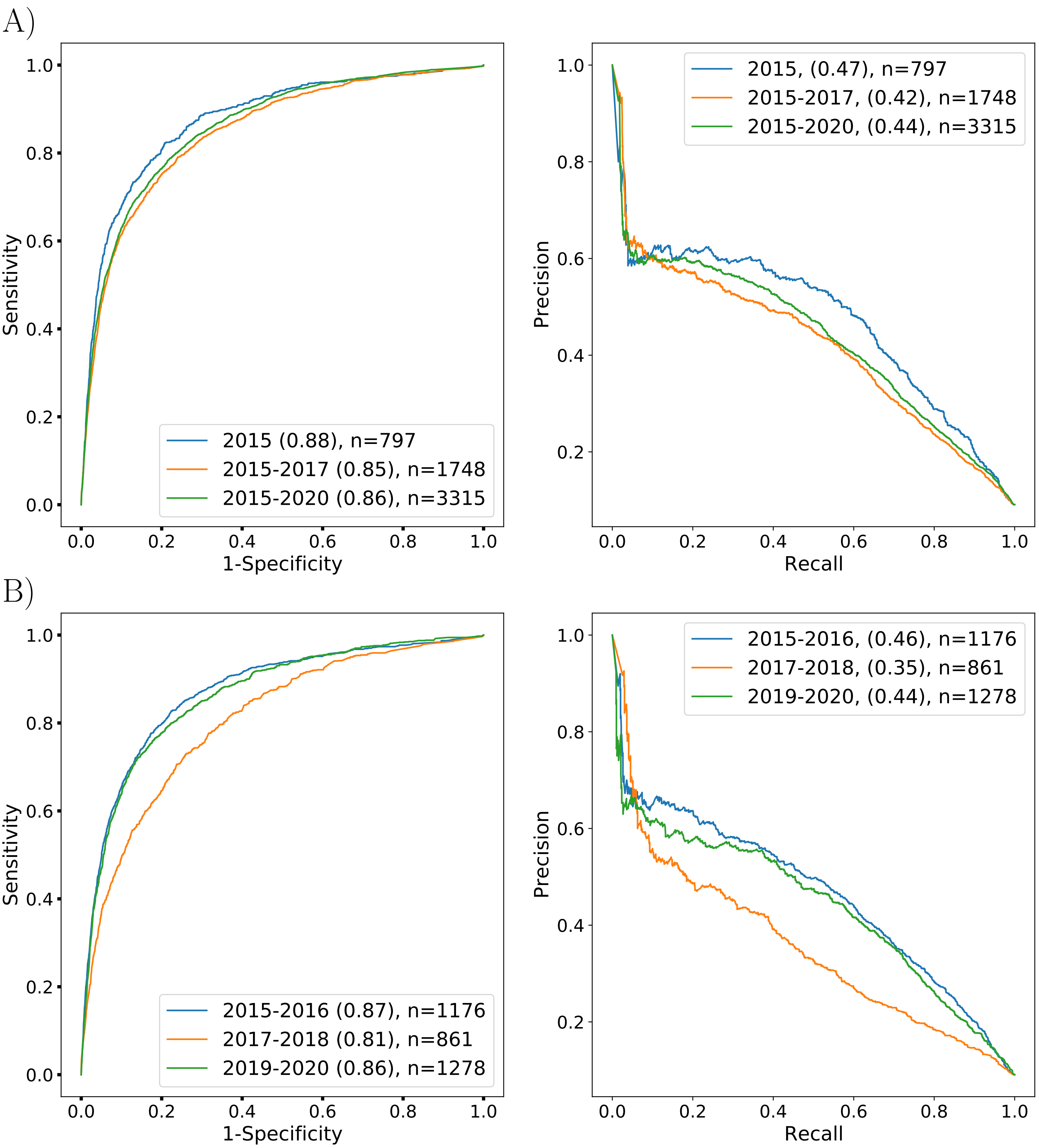

Figure S11. Clinical trials and protein-kinase inhibitors (all phases, 2014, p_ki=10). The left side of each panel shows receiver-operating characteristic analysis (ROC) and the right side shows precision-recall analysis (PR). Training data included abstracts and studies up to 2014. Data from all four phases were included with p_ki=10 (maximum PKs per PKI; see methods). Panels A and B show results with testing data from various periods.

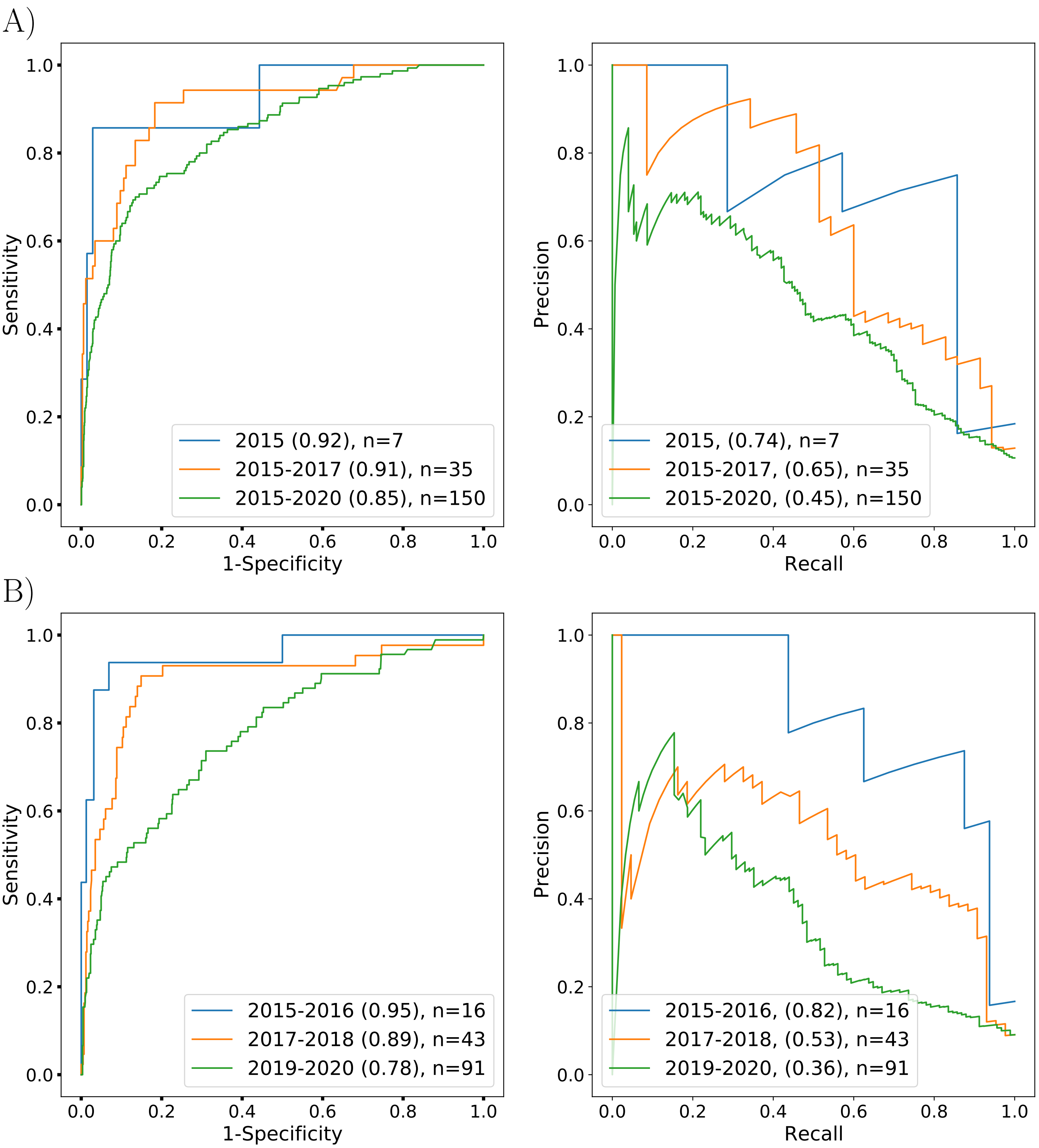

Figure S12. Clinical trials and protein-kinase inhibitors (phase 4, 2014, p_ki=1). The left side of each panel shows receiver-operating characteristic analysis (ROC) and the right side shows precision-recall analysis (PR). Training data included abstracts and studies up to 2014. Data from all four phases were included with p_ki=1 (maximum PKs per PKI; see methods). Panels A and B show results with testing data from various periods.

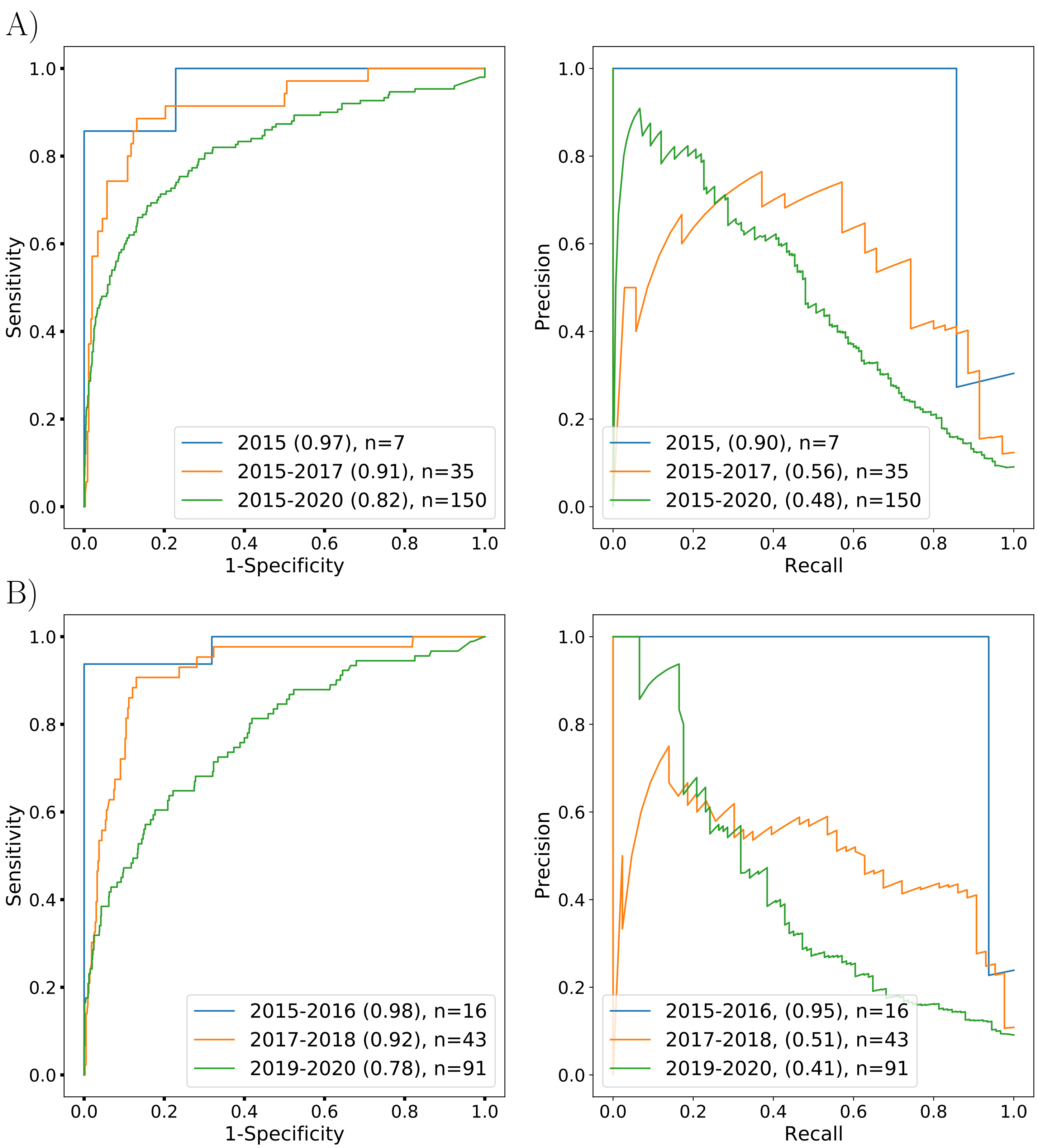

Figure S13. Clinical trials and protein-kinase inhibitors (phase 4, 2014, p_ki=2). The left side of each panel shows receiver-operating characteristic analysis (ROC) and the right side shows precision-recall analysis (PR). Training data included abstracts and studies up to 2014. Data from all four phases were included with p_ki=2 (maximum PKs per PKI; see methods). Panels A and B show results with testing data from various periods.

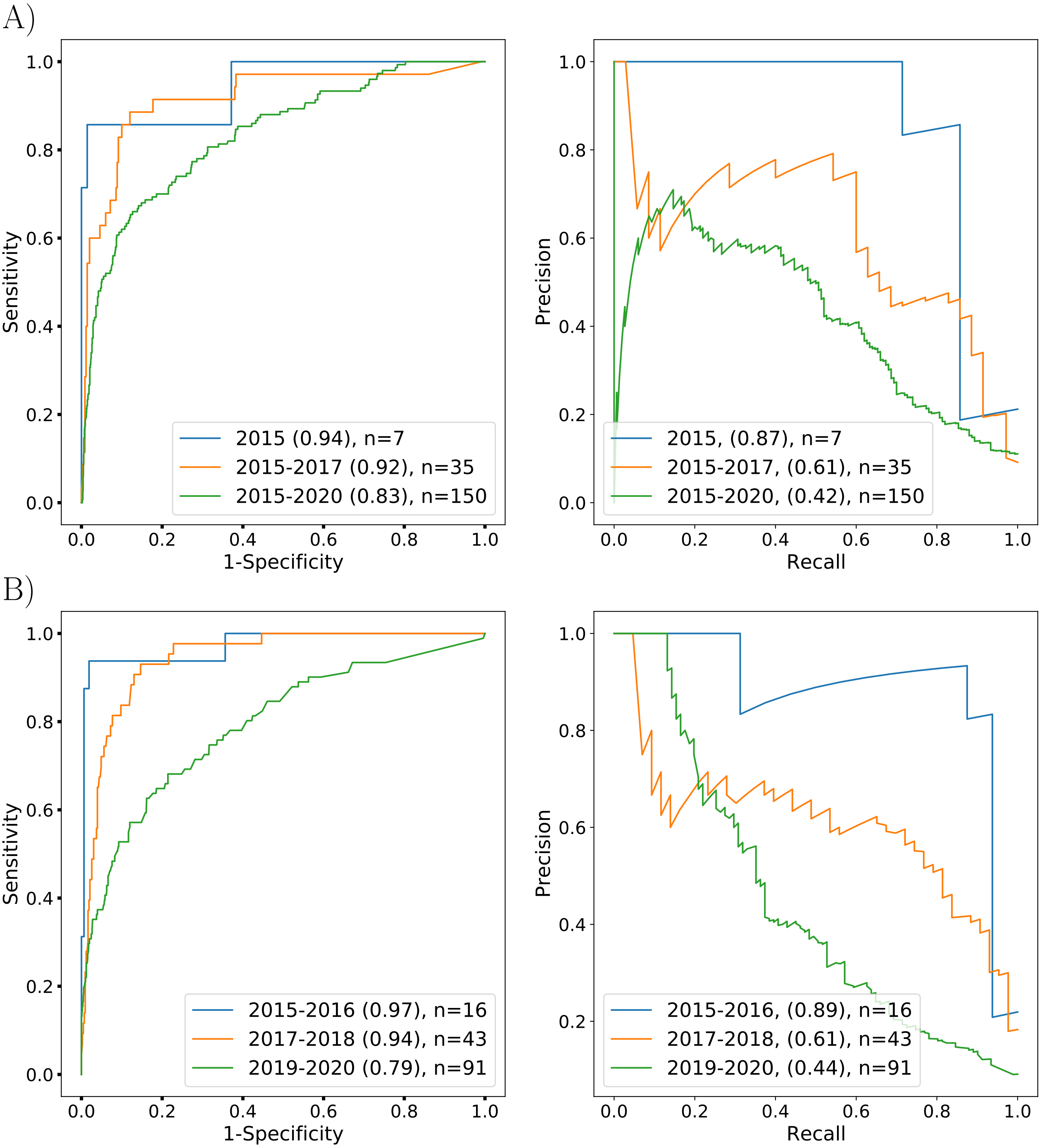

Figure S14. Clinical trials and protein-kinase inhibitors (phase 4, 2014, p_ki=5). The left side of each panel shows receiver-operating characteristic analysis (ROC) and the right side shows precision-recall analysis (PR). Training data included abstracts and studies up to 2014. Data from all four phases were included with p_ki=5 (maximum PKs per PKI; see methods). Panels A and B show results with testing data from various periods.

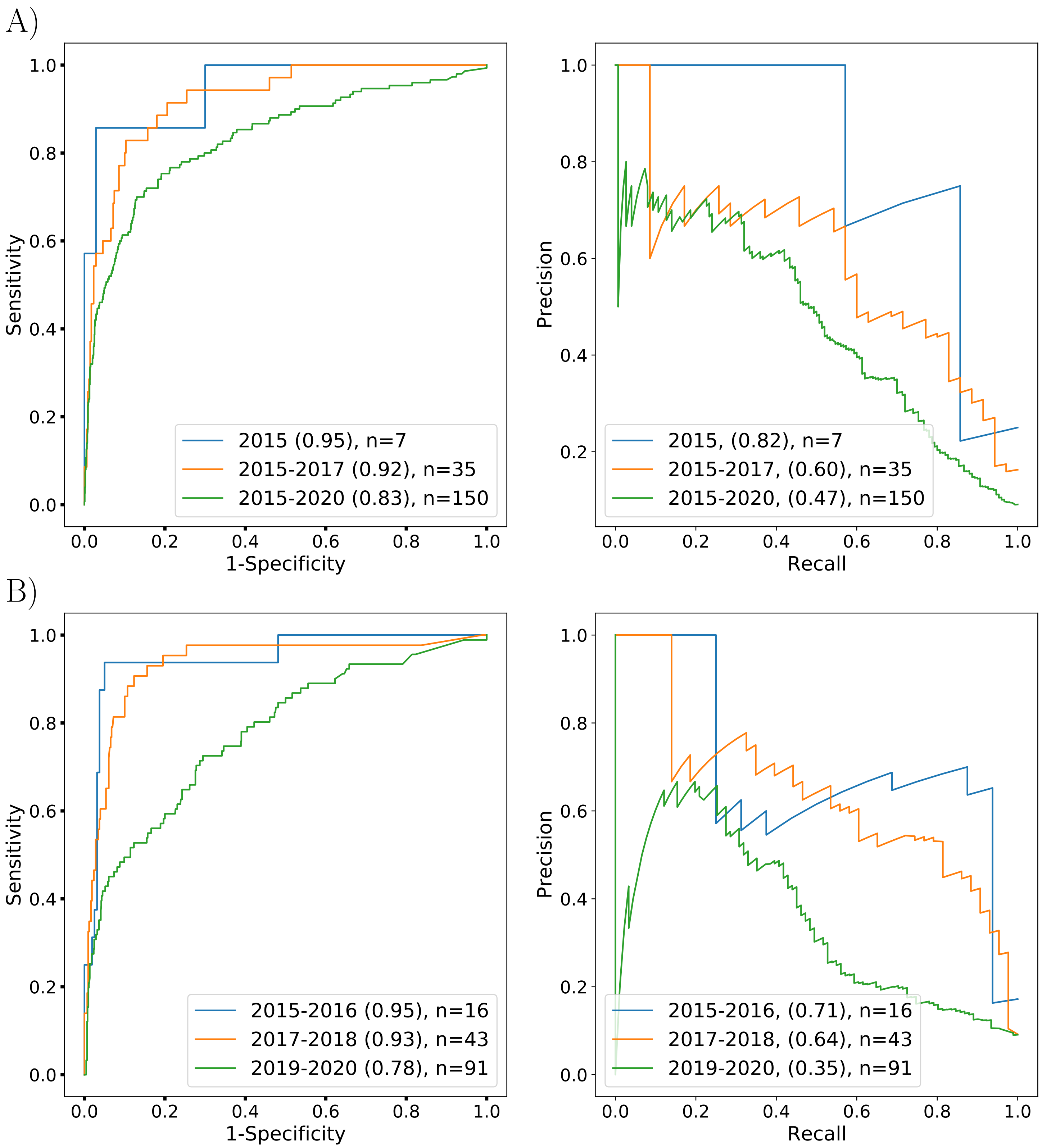

Figure S15. Clinical trials and protein-kinase inhibitors (phase 4, 2014, p_ki=10). The left side of each panel shows receiver-operating characteristic analysis (ROC) and the right side shows precision-recall analysis (PR). Training data included abstracts and studies up to 2014. Data from all four phases were included with p_ki=10 (maximum PKs per PKI; see methods). Panels A and B show results with testing data from various periods.

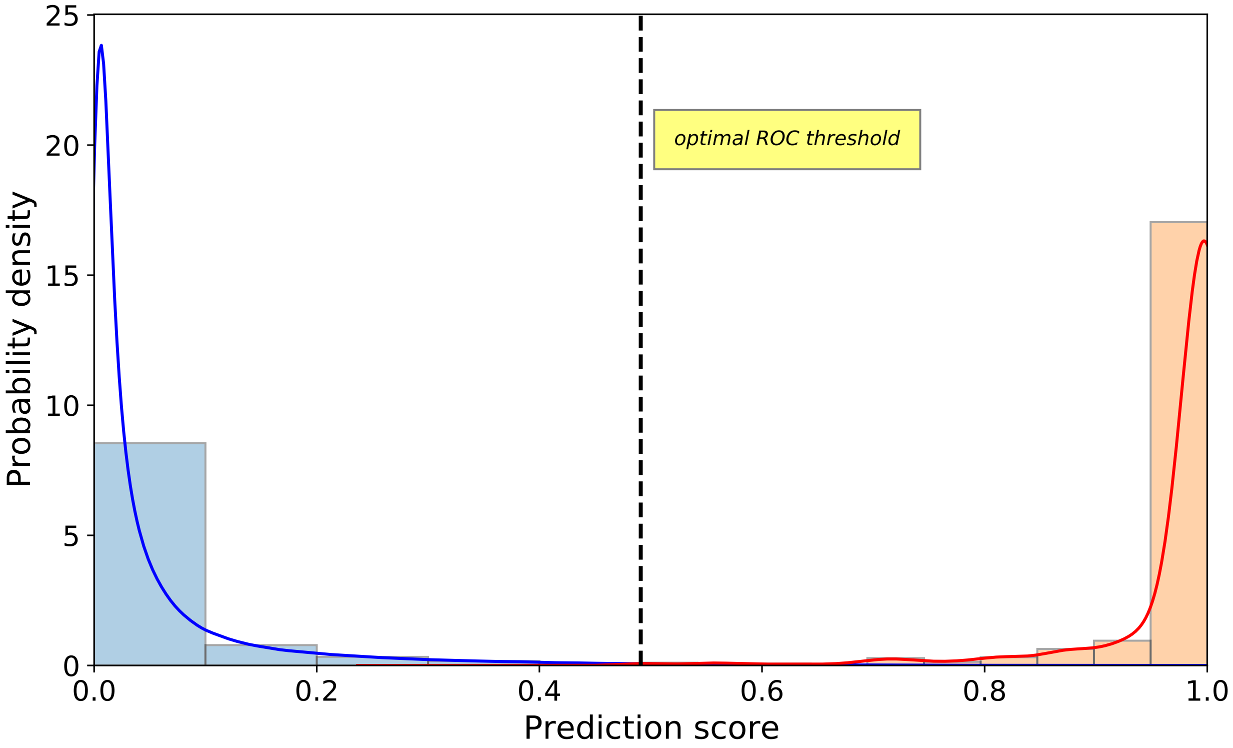

Figure S16. Probability distribution of positive examples and new predictions. We derived concept embeddings from our corpus of PubMed abstracts downloaded in November, 2020. We derived positive links between protein kinases and cancers by considering all clinical trials up to 2020 and also clinical trials that have been verified in 2021. We then constructed the positive training set using all PK-cancer pairs from clinical trials of phase IV. The negative training set contains randomly generated pairs of PKs and cancers where the clinical trials data did not contain any study on using a PKI to treat a cancer, where the PKI inhibits the PK in question (See Methods in manuscript for details). Similar to the historical prediction analysis, we chose the size of the negative training set to be 10 times the size of the positive training set. The figure shows the distribution of probabilities calculated by the random forest model for positive (red) and all other examples.

**Item**	**count**
protein kinases in DrugCentral PKI-PK dataset	68
protein-kinase inhibitors in DrugCentral PKI-PK dataset	79
Relevant abstracts (PubMed 1939 to 2020, with mentions of cancers or protein kinases)	2,779,507
MeSH terms for neoplasms (descendents of D009369)	698
MeSH neoplasms terms identified by PubTator concept replacement in our abstract set	672
NCBIGene ids for kinases	522
Kinases identified by PubTator concept replacement in our abstract set	508
Total PK/cancer pairs considered	508×672=341,376
Total PK/cancer pairs involved in clinical trials up to 2020	7909
Total PK/cancer pairs not yet tested	333,467

Table S1. Summary of data used for training the random forest model.

Years	n_pk	AUROC	Threshold	F-score	Precision	Recall	n
2011-2012	1	0.760	0.175	0.348	0.297	0.414	567
2013-2014	1	0.805	0.115	0.381	0.296	0.526	665
2015-2016	1	0.863	0.195	0.482	0.448	0.515	1176
2017-2018	1	0.789	0.090	0.348	0.253	0.542	861
2019-2020	1	0.849	0.140	0.445	0.369	0.557	1278
2011	1	0.771	0.147	0.355	0.307	0.411	302
2011-2014	1	0.780	0.145	0.365	0.296	0.472	1232
2011-2017	1	0.815	0.138	0.415	0.341	0.528	2980
2011-2020	1	0.823	0.140	0.412	0.338	0.524	4547
2011-2012	2	0.769	0.165	0.341	0.291	0.399	567
2013-2014	2	0.806	0.131	0.391	0.320	0.501	665
2015-2016	2	0.857	0.185	0.463	0.416	0.520	1176
2017-2018	2	0.783	0.102	0.342	0.256	0.512	861
2019-2020	2	0.855	0.150	0.465	0.396	0.560	1278
2011	2	0.767	0.100	0.346	0.259	0.503	302
2011-2014	2	0.787	0.145	0.364	0.314	0.430	1232
2011-2017	2	0.806	0.155	0.391	0.328	0.479	2980
2011-2020	2	0.827	0.145	0.415	0.343	0.525	4547
2011-2012	5	0.767	0.145	0.342	0.285	0.418	567
2013-2014	5	0.802	0.155	0.392	0.330	0.480	665
2015-2016	5	0.856	0.145	0.450	0.368	0.577	1176
2017-2018	5	0.781	0.117	0.347	0.263	0.509	861
2019-2020	5	0.857	0.210	0.467	0.457	0.474	1278
2011	5	0.773	0.155	0.370	0.311	0.437	302
2011-2014	5	0.783	0.175	0.361	0.332	0.394	1232
2011-2017	5	0.817	0.181	0.397	0.359	0.444	2980
2011-2020	5	0.824	0.145	0.404	0.334	0.505	4547
2011-2012	10	0.766	0.135	0.345	0.276	0.459	567
2013-2014	10	0.809	0.140	0.402	0.333	0.487	665
2015-2016	10	0.858	0.170	0.466	0.390	0.571	1176
2017-2018	10	0.767	0.115	0.325	0.253	0.446	861
2019-2020	10	0.859	0.190	0.472	0.441	0.508	1278
2011	10	0.766	0.120	0.339	0.261	0.460	302
2011-2014	10	0.791	0.122	0.364	0.280	0.517	1232
2011-2017	10	0.817	0.145	0.404	0.332	0.513	2980
2011-2020	10	0.833	0.195	0.426	0.385	0.470	4547

Table S2. Summary of results for random forest classification (2010 dataset, all phases). AUROC: area under the ROC curve. n_pk: number of protein kinases per protein-kinase inhibitor. Threshold: Optimal F1 threshold for PR analysis (corresponding to F-Score). Precision/Recall – indicated for the threshold.

Years	n_pk	AUROC	Threshold	F-score	Precision	Recall	n
2011-2012	1	0.898	0.185	0.643	0.516	0.696	23
2013-2014	1	0.946	0.255	0.667	0.667	0.636	22
2015-2016	1	0.874	0.175	0.523	0.415	0.667	33
2017-2018	1	0.892	0.620	nan	0.000	0.000	63
2019-2020	1	0.792	0.765	nan	0.000	0.000	154
2011	1	0.900	0.495	nan	0.000	0.000	4
2011-2014	1	0.929	0.200	0.614	0.566	0.667	45
2011-2017	1	0.894	0.175	0.524	0.416	0.655	113
2011-2020	1	0.861	0.155	0.499	0.415	0.614	295
2011-2012	2	0.957	0.305	0.732	0.812	0.565	23
2013-2014	2	0.925	0.170	0.623	0.500	0.773	22
2015-2016	2	0.889	0.640	nan	0.000	0.000	33
2017-2018	2	0.873	0.640	nan	0.000	0.000	63
2019-2020	2	0.818	0.155	0.449	0.397	0.513	154
2011	2	0.909	0.300	0.667	0.500	0.500	4
2011-2014	2	0.926	0.240	0.638	0.604	0.644	45
2011-2017	2	0.905	0.195	0.576	0.500	0.664	113
2011-2020	2	0.870	0.175	0.511	0.438	0.597	295
2011-2012	5	0.963	0.190	0.708	0.667	0.696	23
2013-2014	5	0.958	0.215	0.739	0.696	0.727	22
2015-2016	5	0.879	0.750	nan	0.000	0.000	33
2017-2018	5	0.863	0.580	nan	0.000	0.000	63
2019-2020	5	0.813	1.000	nan	0.000	0.000	154
2011	5	0.963	0.155	0.727	0.500	0.750	4
2011-2014	5	0.951	0.255	0.646	0.604	0.644	45
2011-2017	5	0.875	1.000	nan	0.000	0.000	113
2011-2020	5	0.869	0.860	nan	0.000	0.000	295
2011-2012	10	0.920	0.205	0.621	0.500	0.696	23
2013-2014	10	0.959	0.785	nan	0.000	0.000	22
2015-2016	10	0.841	0.770	nan	0.000	0.000	33
2017-2018	10	0.866	0.625	nan	0.000	0.000	63
2019-2020	10	0.827	0.830	nan	0.000	0.000	154
2011	10	0.875	0.640	nan	0.000	0.000	4
2011-2014	10	0.919	0.200	0.606	0.500	0.667	45
2011-2017	10	0.876	0.225	0.504	0.462	0.540	113
2011-2020	10	0.865	0.185	0.501	0.447	0.553	295

Table S3. Summary of results for random forest classification (2010 dataset, phase 4). Abbreviations as in Table S2

Years	n_pk	AUROC	Threshold	F-score	Precision	Recall	n
2015-2016	1	0.879	0.184	0.533	0.455	0.640	1176
2017-2018	1	0.819	0.120	0.398	0.320	0.524	861
2019-2020	1	0.864	0.205	0.484	0.447	0.523	1278
2015	1	0.886	0.195	0.557	0.500	0.624	797
2015-2017	1	0.851	0.170	0.468	0.409	0.546	1748
2015-2020	1	0.852	0.175	0.488	0.430	0.562	3315
2015-2016	2	0.876	0.200	0.543	0.501	0.591	1176
2017-2018	2	0.812	0.195	0.402	0.400	0.402	861
2019-2020	2	0.868	0.220	0.494	0.461	0.531	1278
2015	2	0.882	0.225	0.536	0.505	0.568	797
2015-2017	2	0.846	0.165	0.473	0.407	0.564	1748
2015-2020	2	0.853	0.180	0.477	0.424	0.544	3315
2015-2016	5	0.874	0.195	0.522	0.462	0.599	1176
2017-2018	5	0.793	0.175	0.383	0.356	0.413	861
2019-2020	5	0.859	0.215	0.498	0.475	0.523	1278
2015	5	0.877	0.205	0.540	0.494	0.593	797
2015-2017	5	0.850	0.190	0.480	0.441	0.526	1748
2015-2020	5	0.853	0.150	0.470	0.385	0.603	3315
2015-2016	10	0.870	0.191	0.510	0.457	0.577	1176
2017-2018	10	0.805	0.220	0.402	0.417	0.387	861
2019-2020	10	0.863	0.191	0.500	0.444	0.571	1278
2015	10	0.876	0.213	0.540	0.496	0.591	797
2015-2017	10	0.848	0.175	0.482	0.421	0.561	1748
2015-2020	10	0.860	0.210	0.490	0.465	0.516	3315

Table S4. Summary of results for random forest classification (2014 dataset, all phases). Abbreviations as in Table **S2**.

**Years**	**n_pk**	**AUROC**	**Threshold**	**F-score**	**Precision**	**Recall**	**n**
2015-2016	1	0.954	0.390	0.800	0.722	0.812	16
2017-2018	1	0.895	0.250	0.568	0.595	0.512	43
2019-2020	1	0.777	0.775	nan	0.000	0.000	91
2015	1	0.924	0.505	0.800	0.714	0.714	7
2015-2017	1	0.911	0.376	0.632	0.810	0.486	35
2015-2020	1	0.847	0.875	nan	0.000	0.000	150
2015-2016	2	0.980	0.395	0.968	1.000	0.875	16
2017-2018	2	0.918	0.805	nan	0.000	0.000	43
2019-2020	2	0.776	0.265	0.424	0.466	0.374	91
2015	2	0.967	0.533	0.923	1.000	0.714	7
2015-2017	2	0.913	0.815	nan	0.000	0.000	35
2015-2020	2	0.819	0.828	nan	0.000	0.000	150
2015-2016	5	0.973	0.355	0.903	0.929	0.812	16
2017-2018	5	0.944	0.185	0.653	0.588	0.698	43
2019-2020	5	0.792	0.305	0.432	0.556	0.330	91
2015	5	0.945	0.460	0.857	0.833	0.714	7
2015-2017	5	0.916	0.340	0.667	0.741	0.571	35
2015-2020	5	0.834	0.865	nan	0.000	0.000	150
2015-2016	10	0.947	0.350	0.778	0.684	0.812	16
2017-2018	10	0.933	0.165	0.642	0.531	0.791	43
2019-2020	10	0.778	0.790	nan	0.000	0.000	91
2015	10	0.949	0.370	0.800	0.714	0.714	7
2015-2017	10	0.922	0.355	0.615	0.655	0.543	35
2015-2020	10	0.834	0.306	0.506	0.579	0.440	150

Table S5. Summary of results for random forest classification (2014 dataset, phase 4). Abbreviations as in Table S2.

Year	Phase	Category	Median	Minimum	Maximum
2010	all phases	F1 (PR)	0.391	0.325	0.482
2010	phase 4	F1 (PR)	0.621	0.449	0.739
2010	all phases	AUROC	0.805	0.760	0.863
2010	phase 4	AUROC	0.890	0.792	0.963
2014	all phases	F1 (PR)	0.489	0.383	0.557
2014	phase 4	F1 (PR)	0.667	0.424	0.968
2014	all phases	AUROC	0.859	0.793	0.886
2014	phase 4	AUROC	0.917	0.776	0.980

Table S6. Summary of median, minimum, and maximum F1 scores (precision recall analysis) and area under the receiver operating characteristic curve from tables S2-S5.

co-occurrences	precision	recall	F1
1	0.081	0.562	0.142
2	0.122	0.464	0.193
3	0.148	0.410	0.218
4	0.168	0.370	0.231
5	0.185	0.342	0.240
6	0.196	0.316	0.242
7	0.206	0.290	0.241
8	0.217	0.274	0.242
9	0.226	0.260	0.242
10	0.234	0.248	0.241
11	0.241	0.233	0.237
12	0.246	0.219	0.231
13	0.254	0.210	0.230
14	0.260	0.202	0.228
15	0.264	0.193	0.223
16	0.270	0.187	0.221
17	0.275	0.181	0.218
18	0.283	0.175	0.216
19	0.286	0.170	0.214
20	0.290	0.165	0.211
21	0.293	0.160	0.207
22	0.295	0.153	0.201
23	0.299	0.149	0.199
24	0.303	0.145	0.196
25	0.303	0.141	0.192

Table S7. Precision recall analysis for baseline co-occurrence analysis (All phases). This analysis was performed as a baseline against which the advantage of our word2vec/random forest approach can be assessed. We counted co-occurrences of the same protein kinase and cancer concepts as used in the main analysis (abstracts up to 2015). If a given pair was found to co-occur at least the indicated number of times (1-25), it was classified as positive, otherwise as negative. The maximum F1 score was 0.242.

co-occurrences	precision	recall	F1
1	0.008	0.693	0.015
2	0.012	0.598	0.024
3	0.015	0.539	0.030
4	0.018	0.502	0.034
5	0.020	0.477	0.039
6	0.022	0.448	0.041
7	0.025	0.448	0.047
8	0.027	0.432	0.050
9	0.029	0.423	0.054
10	0.030	0.411	0.056
11	0.032	0.398	0.059
12	0.034	0.390	0.063
13	0.036	0.378	0.065
14	0.037	0.365	0.067
15	0.038	0.353	0.068
16	0.040	0.353	0.071
17	0.041	0.344	0.073
18	0.043	0.340	0.076
19	0.044	0.336	0.078
20	0.045	0.328	0.079
21	0.046	0.320	0.080
22	0.048	0.320	0.084
23	0.049	0.311	0.084
24	0.050	0.307	0.086
25	0.051	0.303	0.087

Table S8. Precision recall analysis for baseline co-occurrence analysis (Phase 4). This analysis was performed as a baseline against which the advantage of our word2vec/random forest approach can be assessed. We counted co-occurrences of the same protein kinase and cancer concepts as used in the main analysis (abstracts up to 2015). If a given pair was found to co-occur at least the indicated number of times (1-25), it was classified as positive, otherwise as negative. The maximum F1 score was 0.087.

References

[1] Chih-Hsuan Wei, Alexis Allot, Robert Leaman, and Zhiyong Lu. Pubtator central: automated concept annotation for biomedical full text articles. *Nucleic acids research*, 47:W587–W593, July 2019.
